# A Multicamera Classroom Dataset for Deep Learning-Based Student Action Recognition and Engagement Analysis

**DOI:** 10.3390/s26206608

**Published:** 2026-10-19

**Authors:** Murilo Cruz Lopes, Ana Paula Serafim de Góis, Aurélio Provetti, Cristiani Vilela Ribeiro Guimarães, Douglas do Prado Pereira, Felipe Augusto Pereira de Figueiredo, Jose Marcos Camara Brito

**Affiliations:** 1 Inatel Competence Center (ICC), Santa Rita do Sapucaí 37540-000, MG, Brazil; 2 Wireless and Artificial Intelligence Laboratory (WAI Lab), Santa Rita do Sapucaí 37540-000, MG, Brazil; 3 National Institute of Telecommunications (INATEL), Santa Rita do Sapucaí 37540-000, MG, Brazil

**Keywords:** classroom, database, neural network, engagement, video clip, behaviour

## Abstract

Classroom behavior analysis has become a relevant area for developing intelligent educational environments, enabling continuous, non-intrusive understanding of student engagement and interactions. In this work, we present a new video clip dataset for recognizing student behavior in the classroom, based on behavior derived from observational protocols. This new dataset also has the feature of obtaining student actions through four different cameras that are positioned frontally and laterally in the classroom. The dataset is then used to train state-of-the-art models for video behavior classification, including Multiscale Vision Transformer (MViTv2-S), Swin Transformer, SlowFast, and X3D. Our results show that MViTv2-S has better performance when accuracy and F1-score are analyzed. The results indicate an accuracy of 74.44 ± 0.45 for the frontal cameras and 70.88% ± 1.04 for the lateral cameras, demonstrating the effectiveness of both camera configurations for student action recognition. When engagement is evaluated by aggregating the classified actions, an accuracy of 83.33% and a macro F1-score of 80.42% are achieved. The results highlight the potential of deep learning-based approaches for classroom behavior analysis, offering insights that may support applications in pedagogical support, student engagement assessment, and the development of interpretable and scalable intelligent educational systems.

## 1. Introduction

The pursuit of novel teaching strategies has spurred the growth of intelligent education, which is recognized as a pivotal pathway to the future of learning. Smart classrooms represent a fundamental component of this process, integrating automation, data analytics, and digital technologies to support instructors and students. Among indicators of teaching quality, student behavior in the classroom is a relevant source of information about their interest, engagement, and learning.

The assessment of classroom student engagement is commonly conducted using manual instruments, including observations, questionnaires, and self-report scales [1,2]. Ref. [3] categorizes engagement estimation methods into three types: manual, semi-automatic, and automatic. Manual methods encompass paper-based, computer-based, or self-reporting [4] questionnaires, such as the National Survey of Student Engagement [5] and the Student Engagement Instrument [2], as well as observational protocols, as described [1,6]. While these methods provide valuable information, their application presents practical challenges because observations are conducted at discrete time intervals; capturing all behavioral variations that occur throughout a lesson, especially in large classes, becomes difficult. Furthermore, because these results are based on human perception, they tend to reflect subjective differences among observers, which can affect the consistency of the analyses. With advancements in artificial intelligence, particularly deep learning and computer vision, it has become possible to automate this process. The use of deep neural networks enables action detection, recognition of behavioral patterns, and the provision of continuous metrics of engagement and attention in interactive environments.

Despite recent progress, real-time behavioral analysis in educational settings still faces significant challenges, including the simultaneous tracking of multiple individuals, ensuring robustness in complex scenarios, reducing model computational costs, and enhancing interpretability. To address these limitations, various studies have explored solutions in computer vision [7,8]. In [9], for example, an intelligent system was developed to monitor student engagement via facial expression recognition. Other works propose innovative approaches for continuous activity recognition, framing the problem as multiple object tracking (MOT). Furthermore, new datasets have been introduced to improve action detection in classrooms, as presented in [10] and also in [11,12], which provide specialized datasets tailored for student behavior recognition.

Given the practical limitations of manual approaches and recent advances in computer vision for engagement monitoring, this work proposes a new dataset, grounded in established classroom observation protocols such as BOSS and MS-CISSAR [6], adapted to the Brazilian higher education context, and built using a robust multicamera dataset. Using this dataset, deep learning models such as MViT [13] were trained and evaluated on existing architectures. The proposed approach utilizes real-world videos from university classrooms, ensuring greater relevance to the educational setting. This strategy enables a comparative evaluation of architectures in an environment representative of actual classroom conditions and pedagogical demands. The principal contributions can be summarized as follows:

- Development of a real-world dataset: Comprising a total of 42,036 video clips of 2.13 s each, spanning multiple sessions and diverse student cohorts across different academic days.
- Definition of classroom behavior categories: Based on observational protocols adapted for higher education, categories include paying attention, using a mobile phone, using a computer, reading, writing, and moving around.
- Systematic evaluation of models: Identification of architectures exhibiting the best performance in classifying these behaviors, providing comparative metrics for model selection.
- Extraction of interpretable engagement metrics: Derived from detected behaviors to enable data-driven analysis of student attention and participation in classroom environments.

This article presents a multicamera classroom dataset designed to support deep learning-based student action recognition and engagement analysis. Initially, a review of related work examines existing research utilizing training and dataset construction for action classification, person detection, student behavior analysis, and classroom engagement. Subsequently, the methodology outlines the definitions and methods employed to identify and categorize key engagement behaviors for model training. The dataset creation process is detailed, including camera placement strategies, dataset partitioning for training and validation, and the distribution of video samples across different behavior classes. The models selected for comparison are also described. The results section presents performance metrics, including accuracy, F1-score, and AUC-ROC, with a comparative discussion of the models’ effectiveness. The conclusion summarizes key findings, reflects on the research process, and proposes avenues for future work. Finally, ethical considerations are addressed, outlining adherence to relevant legal guidelines regarding data handling and privacy.

## 2. Related Work

Numerous studies have explored the use of computer vision and machine learning to understand student engagement and behavior in the classroom. This section discusses the key works that underpin this research, grouped by methodological approach.

The study by [9] proposed a system for monitoring student engagement in physical classrooms. The authors describe a methodology focused on processing videos captured in the educational environment to analyze student engagement levels (low, medium, and high) and present them to the instructor in real time. The methodological framework comprises two distinct modules: an offline module and an online module.

The offline module is responsible for training a convolutional neural network-based facial expression recognition (FER) model. The BAUM-1, DAiSEE, and YawDD datasets were utilized for this training. The online module, conversely, operates in real time, employing the pre-trained model to estimate student engagement during classes.

The trained model achieved 78.70% accuracy on the training dataset and 76.90% on the validation dataset. It was developed to classify six affective states (sleepy, yawn, boredom, frustration, confusion, and focus) using images from the CSFED dataset, comprising approximately 1193 distinct student faces obtained from both real classroom recordings and supplementary public datasets.

In the results, the model achieved 76.9% accuracy, 77% average precision, and 76% sensitivity, with the best performance observed for the yawning and sleepy classes. Furthermore, the correlation between automatic predictions and self-reported engagement levels (Group Engagement Level—GEL) was 73.68%, demonstrating the method’s viability for automated, real-time student engagement analysis.

Automatic recognition of human actions and behaviors (Human Action Recognition—HAR and Human Behavior Recognition—HBR) has advanced significantly in educational research focused on student engagement analysis. In the study by [10], the following classes were used for model training: raising hand, using phone, sleeping, standing, and writing. The CStudentAct dataset comprises 55,189 annotated bounding boxes, enabling various experiments with different detection architectures, including YOLOv5n, YOLOv5s, YOLOv5m, YOLOv5l, and RT-DETR (R50). The best results were observed for the sleeping (0.94 mAP50) and standing activities (0.905 mAP50) classes, which demonstrated superior performance compared to actions such as writing (0.827 mAP50) and using a phone (0.584 mAP50). These results indicate that for more complex actions, such as using a mobile phone frequently characterized by severe occlusions, automatic recognition can be substantially improved through more robust detection and modeling techniques.

The study proposed by [14] integrates ResNet-18, the Vision Transformer (ViT), and MSSTGCN architectures to detect actions performed by students and instructors during class periods. Instructor actions are categorized as writing on the blackboard, front-standing lecture, side-standing lecture, and inspecting the classroom. At the same time, students are classified into six behaviors: listening, reading, writing, playing with mobile phones, sleeping, and talking. The dataset utilized comprises 4500 videos, with durations ranging from 8 to 10 s. The model was implemented in PyTorch 2.5.1, using the TV-L1 method for optical flow estimation between adjacent frames. The results demonstrate an accuracy of 90% in classifying instructor actions, with inspecting the classroom (98.4%) and side-standing lecture (92.6%) exhibiting the highest performance. For student action recognition, the MSSTGCN model achieved the best accuracy, reaching 87%. The authors also conducted a comparative analysis of instructor and student behaviors during the same classes and coincident periods of the academic session. The framework-based method combining ResNet-18 and ViT across the RGB + Optical Flow modalities achieved the best overall accuracy of 95.5%.

Additionally, the MSTA-SlowFast model [11], which focuses on detecting seven common classroom behaviors, such as looking at the board, raising a hand, lying on the desk, talking, and tilting the head, and providing information about student positions and actions, was employed as a base architecture in this stage. Within this model, MSTA (multiscale spatial-temporal attention) blocks were incorporated into SlowFast to extract spatial information at multiple scales and establish long-range channel dependencies. To complement this, ETA (efficient temporal attention) blocks were introduced to enhance temporal attention calculation. The dataset used consists of 478 videos, annotated in 80 categories, with an annotation rate of 1 frame per second. Approximately 250 min of classes were annotated, predominantly from the frontal angle of the classrooms. Among the experiments conducted, the SlowFast + MSTA + ETA combination achieved the best performance, with a recall of 81.4%, a precision of 81.9%, and an mAP of 91.10%. These results demonstrate that incorporating spatial–temporal attention mechanisms achieved significant advances in educational behavior recognition, resulting in a 5.63% improvement over the original model.

Among datasets focused on classroom behavioral analysis, EduNet [15] stands out. This dataset comprises approximately 20 action categories in an educational environment, totaling 7,851 videos with durations ranging from 3 to 12 s, with at least 200 clips per class. The actions are organized into two groups:
**Teacher-centric labels:****Student-centric labels:**Explaining the subject;Hitting;Holding book;Holding mobile phone;Holding sticks;Sitting on chair;Slapping;Walking in the classroom;Writing on board.Arguing;Clapping;Eating in the classroom;Gossip;Hand raise;Reading book;Sitting on desk;Sleeping;Standing;Talking;Writing on textbook.

For action recognition, the authors used the pre-trained I3D-ResNet-50 model on the Kinetics-400 dataset, fine-tuning it to adapt it to EduNet. While the model achieved 97.8% accuracy on the original dataset, its performance on EduNet dropped to 72.3%, indicating that the dataset is more complex and variable, which complicates generalization.

Numerous studies have demonstrated the potential of computer vision for action recognition and behavioral analysis in educational environments, contributing to an understanding of student engagement, see Table 1. Building on these advancements, the present work continues this line of investigation by proposing improvements to data collection, annotation, and representation processes. The methodology distinguishes itself through its application in a real-world university setting, of greater scale and complexity, utilizing multiple cameras and a continuous classroom recording with selective spatiotemporal annotation. Furthermore, new behavioral categories have been incorporated to reflect different manifestations of engagement and disengagement, such as paying attention and *moving around*, enabling inference of the student’s level of involvement from observable actions. The proposed method is also less intrusive, as it performs analysis automatically without interfering with the natural classroom dynamics. This integrative approach combines detection, tracking, and categorization techniques to provide a more precise, contextually informed interpretation of student behavior.

## 3. Methodology

This section describes the construction of the multicamera classroom dataset designed to support deep learning-based student action recognition and engagement analysis. The process of data collection, selection, and organization of samples is described, along with the parameters adopted to define the behavioral classes that contribute to the formation of an observational engagement profile. Data collection was conducted at a higher education institution, conferring diverse characteristics of interaction between students and instructors to the setting. The observed actions vary according to the academic context, encompassing both behaviors of seated students, such as writing, using a computer, and actions of movement, such as walking around the room. In the subsequent sections, the criteria for prioritization and standardization of these classes are detailed, which are fundamental for the model training stage. Following this, the neural networks chosen for training using the proposed dataset and the hyperparameters selected for training are described.

### 3.1. Definition of Engagement and Its Dimensions

The definition of engagement adopted in this work is based on the concepts presented by [2,5,6,16], according to which engagement corresponds to the degree of emotional and behavioral involvement of the student during teaching–learning activities. As the authors emphasize, engagement cannot be accurately assessed solely through direct observation or large-scale questionnaires; rather, it is more appropriate to estimate this involvement through the analysis of affective states, such as facial expressions, gestures, and body postures, captured in classroom recordings. Thus, engagement was not measured directly or predicted by deep learning models; instead, it was indirectly estimated from observable behaviors that served as behavioral proxies for the corresponding engagement levels.

Based on this perspective, the present study adopts a context-specific operationalization of the engagement categorization proposed by [9], structuring engagement into three primary levels according to the observable behaviors and classroom context represented in the dataset:

- Low Engagement (EL1): Characterized by behaviors of inattention or disinterest, such as boredom, sleepiness, and a wandering gaze. This level includes actions such as
  − Moving;
  − On phone.
- Medium Engagement (EL2): Associated with states of cognitive effort accompanied by distraction or frustration, indicating an oscillating level of participation. This group includes actions such as
  − On computer.
- High Engagement (EL3): Defined by behaviors that demonstrate focus and concentrated attention, revealing active involvement with the activities. Examples of actions at this level include
  − Paying attention;
  − Writing;
  − Reading.

These levels were used as a reference for categorizing the behavioral actions observed in the dataset, enabling the recognized behaviors to be interpreted in terms of different levels of student engagement based on the visual evidence collected in the classroom. Recognizing student behavior represents an important initial step toward classifying engagement into the three levels (EL1–EL3), particularly because engagement is context-dependent. For example, a behavior such as moving may be interpreted differently depending on the classroom context and activity. Thus, recognizing observable behaviors first allows their mapping to the engagement levels to be adapted according to the specific context. Thus, EL1–EL3 represent behavior-derived engagement levels rather than independently annotated engagement labels.

### 3.2. Recording Configuration

**Figure 1:**
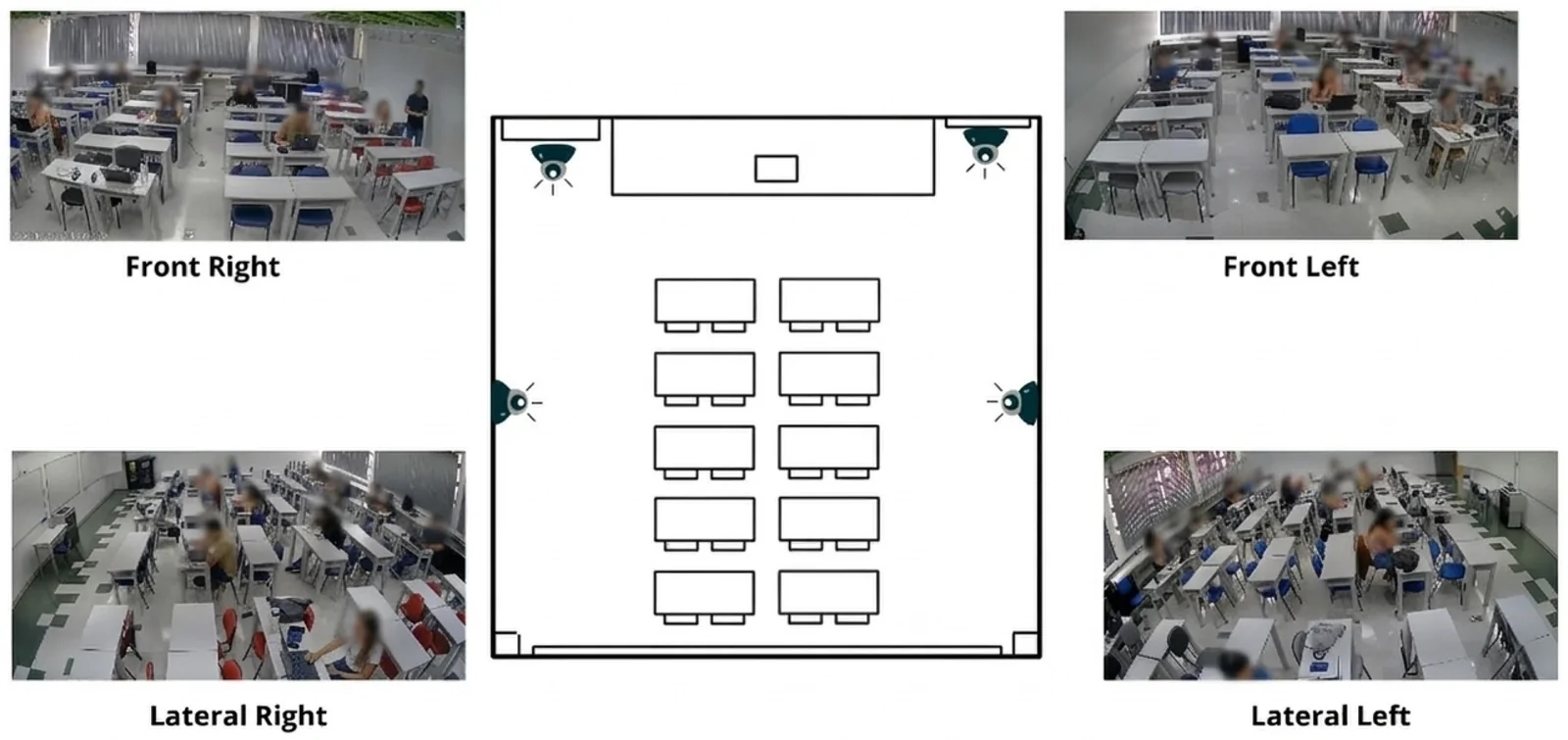
Arrangement and angles of the cameras in the classrooms, with real examples of the captures.

To comprehensively capture participant behavior, recordings were made using the high-definition Intelbras iM5 Full color of resolution Full HD (1080p) (https://www.intelbras.com/pt-br/camera-externa-inteligente-wi-fi-full-color-im5-full-color (accessed on 27 September 2026)), positioned at four strategic points in the classroom, Figure 1. This configuration, with two frontal cameras and two lateral cameras, aimed to ensure spatial coverage of the room and obtain multiple perspectives for subsequent annotation. The intentional overlap of each camera’s field of view was implemented to minimize occlusions, a significant challenge in behavior detection in classroom environments [10,17,18], and preserve the naturalness of participant behavior.

In contrast to methodologies that rely on isolated video snippets, recordings were conducted continuously throughout the duration of the classes. This approach provides a more comprehensive temporal representation of student behavior and enables the analysis of changes in actions over time. With these metrics in perspective, we were able to strategically identify the specific actions and video segments that warranted annotation. This ensured that the labeled data captures the critical moments when students enter or exit different states of involvement. Furthermore, conducting sessions during different periods (afternoon and evening) enabled the observation of variations in these transitions, as well as differences in class composition and discipline dynamics.

### 3.3. Annotation Protocol

**Figure 2:**
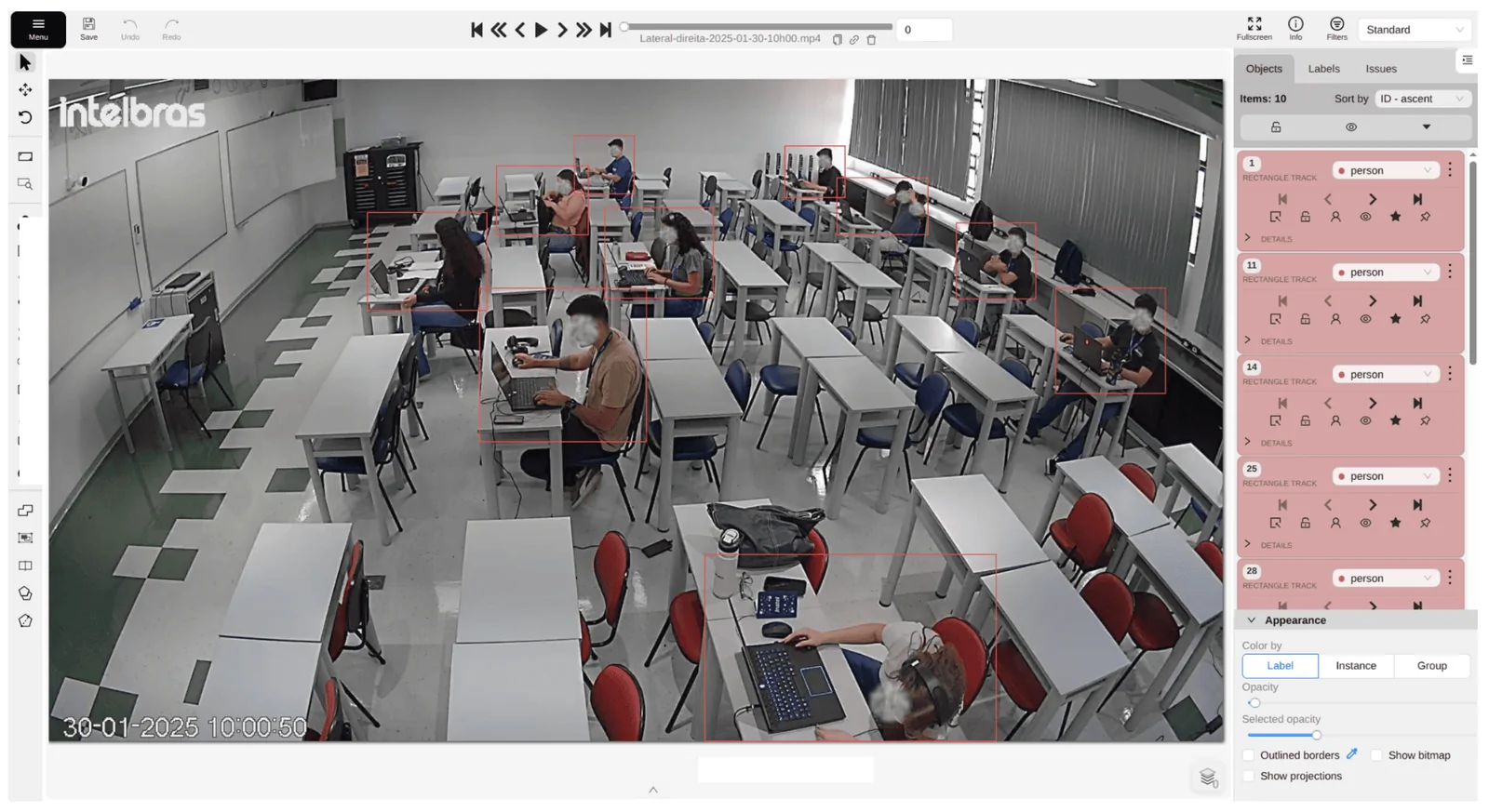
Example of the student annotation process in CVAT (Computer Vision Annotation Tool).

The raw dataset consists of 468 h of footage, corresponding to the total duration of class sessions recorded over two academic semesters. Each video file represents a discrete 90 min session, captured in real-time to align with the natural instructional period. This four-camera setup ensures a comprehensive multi-angle view of the classroom environment. To facilitate systematic analysis, the footage was processed through *CVAT* (Computer Vision Annotation Tool), where individual students were meticulously labeled to capture their positioning and interactions. As illustrated in Figure 2, this annotation workflow allowed for the standardization of samples across the large-scale dataset, providing a clear visual representation of student activity within the classroom space.

The continuous recordings were screened to identify segments containing instances of the predefined behavioral classes. These segments were then extracted and annotated. Because the classroom recordings consisted of approximately 90 min classes of continuous observation, annotating the entire recordings would have produced a large number of highly redundant samples. Students frequently maintained the same behavior for extended periods, such as remaining seated and facing the front of the classroom or continuously using a computer. Consequently, consecutive samples from the same recording could contain essentially identical visual and behavioral information, substantially increasing the dataset size without providing a proportional increase in behavioral diversity.

Once imported into *CVAT*, each student was individually hand-tracked through a frame-by-frame annotation process. Each bounding box on *CVAT* has an *id* that remains on it for several frames until its occlusion by the annotator. This ensured that bounding boxes remained consistent and precisely centered on the student throughout the temporal sequence. The bounding boxes for each student encompass the student’s body and a portion of the student’s desk, including school materials with which the student interacts, such as the computer and notebooks, as illustrated in Figure 2.

**Figure 3:**
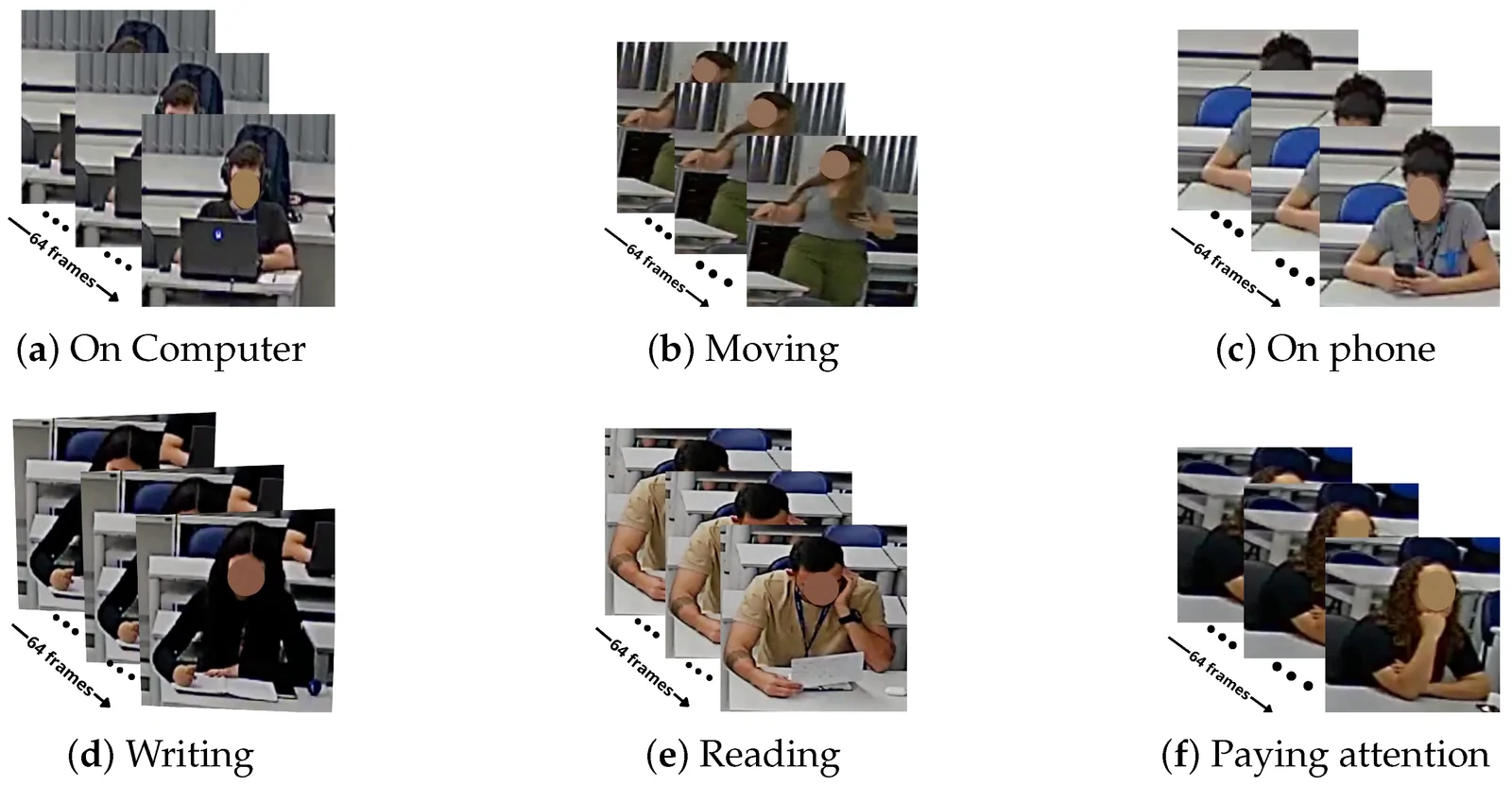
Examples of actions performed by students in the classroom from the frontal camera.

**Figure 4:**
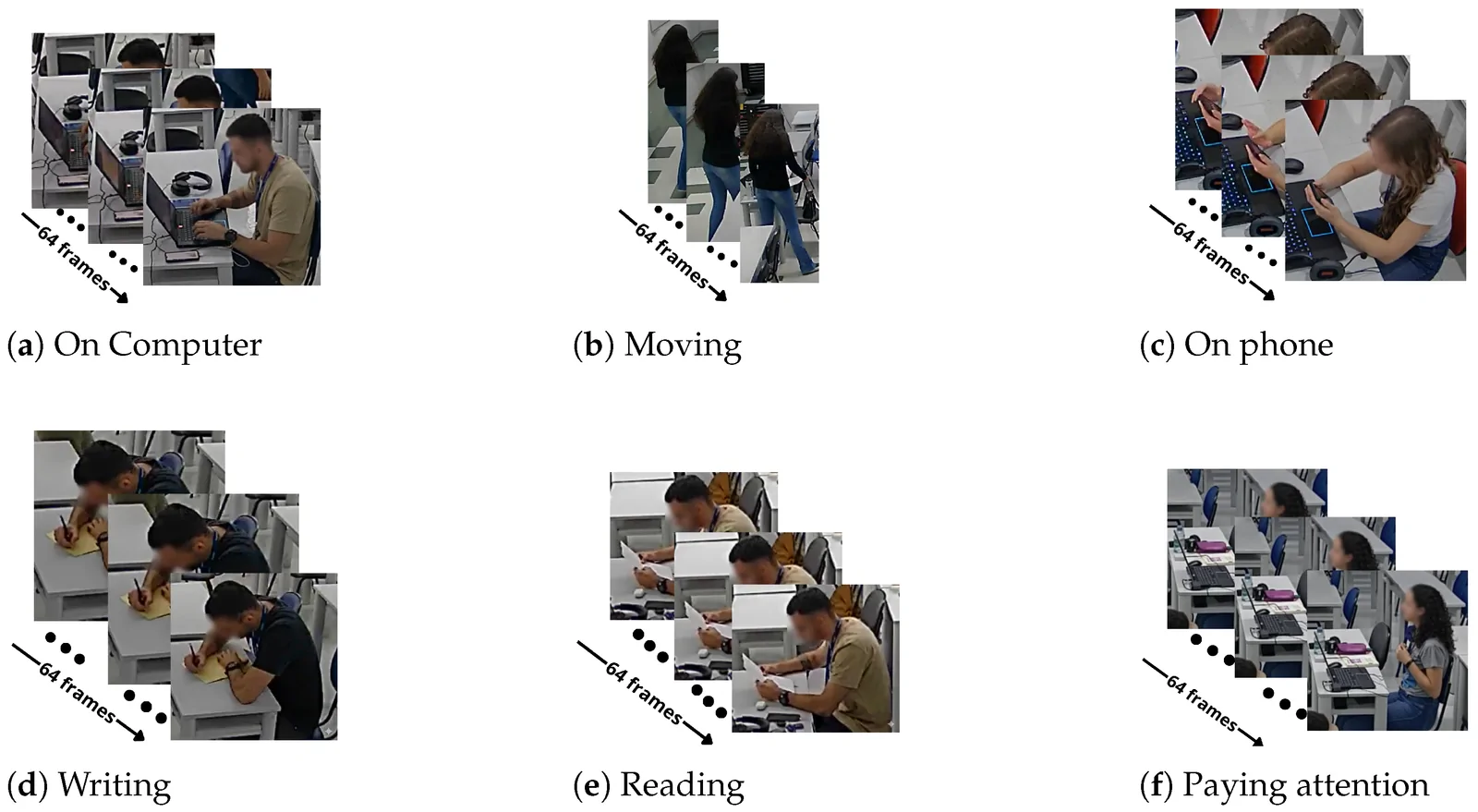
Examples of video clips of the actions performed by students in the classroom from the lateral camera.

The final stage utilized a custom Python 3.10.12 script to transform these annotated segments into a video clip for each student behavior class. The script parses the *CVAT* for a video file, a *.xml* file that contains all the annotation information metadata to perform precise spatial crops on each participant, effectively isolating individuals from the classroom background. This process generates standardized 2.13 s clips at 30 FPS, with each clip containing 64 frames of a student action. As illustrated in Figure 3 and Figure 4, this converts the wide-angle classroom view into a specialized gallery of individual actions. Finally, the script automatically organizes these clips into a directory hierarchy based on the behavior classes assigned in *CVAT*, resulting in a fully categorized dataset ready for model training.

The actions considered in this work include:

- On computer;
- Moving;
- On phone;
- Writing;
- Reading;
- Paying attention.

Figure 3 and Figure 4 provide a visual representation of some video clips for each class of the dataset, showcasing both frontal and lateral camera perspectives of the annotations performed for various behavioral actions. These samples demonstrate not only the consistency of the bounding boxes across different subjects but also the diversity of the captured classroom scenarios. It is important to note that the recordings were made using static cameras installed at fixed positions throughout the semester. These devices remained stationary, without the use of pan, tilt, or zoom (PTZ) movements, to ensure a consistent field of view for analysis over time. Therefore, no preprocessing was performed at this stage; only the capture and annotation of the videos were carried out.

Each clip was subsequently reviewed by a second annotator, and any discrepancies were resolved by consensus, ensuring inter-annotator consistency. One point of the review was to compare writing on a tablet to the act of writing, and reading on phone to reading on a phone. When a student was using a calculator, it was categorized as on phone, due to the difficulty in distinguishing it from a certain distance from the student to the camera. These decisions were made because they occurred occasionally. Another situation is when students are positioned further from the camera or in areas with limited visibility, ambiguity can arise in interpreting their actions. This is occasioned by the traditional classroom arrangement of students seated in rows, which often results in occlusion where one student’s desk obstructs the view of another. To mitigate potential data ambiguity and complexity, annotations for significantly occluded students were excluded from the dataset. In case of data ambiguity, the annotation focuses on the action that is visually predominant or most clearly observable within the clip, rather than attempting to represent all concurrent behaviors with a single label. However, no quantitative inter-annotator agreement measure was calculated.

### 3.4. Dataset

**Figure 5:**
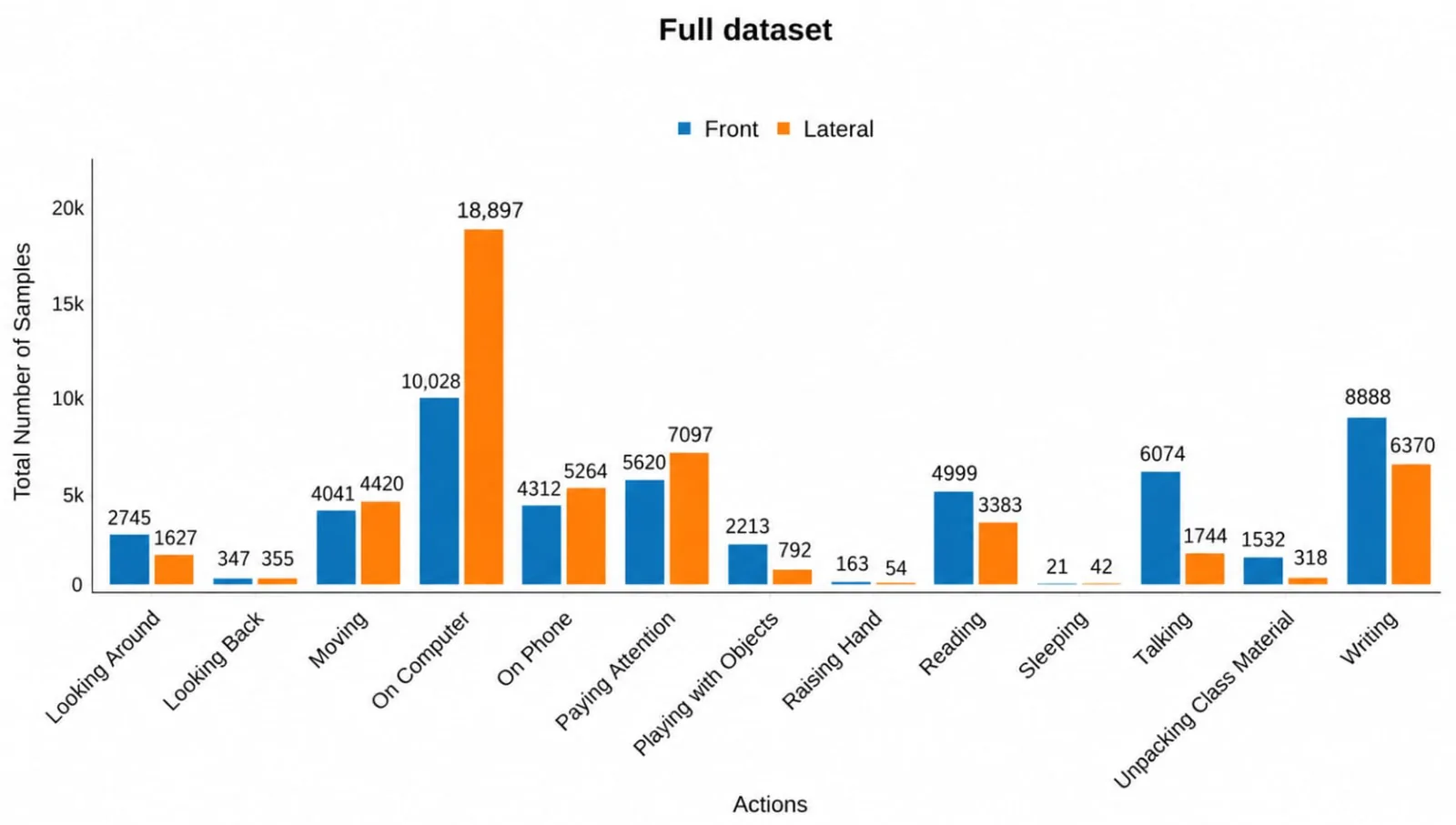
Graph of the quantity of videos per action for the frontal and lateral cameras.

The dataset was developed to comprehensively capture student behaviors within a classroom environment, focusing on actions indicative of classroom engagement. Recordings were conducted across diverse classes, incorporating variations in student numbers (ranging from 10 to 50 students per classes), lighting conditions, gender, varying ages (18 to 45 years) and classroom layouts to accurately reflect realistic learning environments. Figure 5 presents the distribution of samples per activity class within the final dataset. The systematic annotation resulted in a final dataset of 101,346 standardized 2.13 s video clips; from the total of 101,346 video clips, 50,363 are from lateral and 50,983 from frontal perspectives. Through Figure 5, it is possible to observe a higher concentration of actions within the classes: on computer, moving, writing, reading, paying attention, and on phone. Several other classes show a smaller quantity of actions, such as sleeping, raising hands, and looking back. The volume of data for each class was strategically managed to ensure a balanced distribution, using the most representative class containing approximately one thousand samples as a benchmark.

It is important to note that the initial annotation phase was more comprehensive than the current training set. A broad range of behaviors was annotated, including looking around, looking back, playing with objects, raising hands, sleeping, talking, and unpacking class material. However, due to time constraints and the limited number of annotators, these classes could not be expanded with as many samples as the selected ones. Therefore, to simplify the initial implementation and achieve higher model accuracy and performance, the focus was narrowed to a specific subset of these classes: on computer, moving, on phone, writing, reading, and paying attention. While the excluded actions remain part of our raw annotated repository, they were omitted from the current training stage to prioritize dataset quality. This approach establishes a robust foundation for the model, while leaving the integration of these additional classes, such as the less frequent behaviors of raising a hand or sleeping, as a goal for future work to further enrich the system’s predictive power.

### 3.5. Dataset Split: Training, Validation, and Test

The train–test split procedure is used to estimate the performance of a machine learning algorithm in making predictions on data not used to train the model. Additionally, a validation set is a sample of data reserved from training the model to estimate the model’s capabilities while the model’s hyperparameters are adjusted, and the capabilities of the final tuned model are evaluated by comparing or selecting between final models. Following current principles of dataset splitting, the samples of each class were divided into training, validation and a test set, with proportions of approximately 70%, 15%, and 15%, respectively.

**Figure 6:**
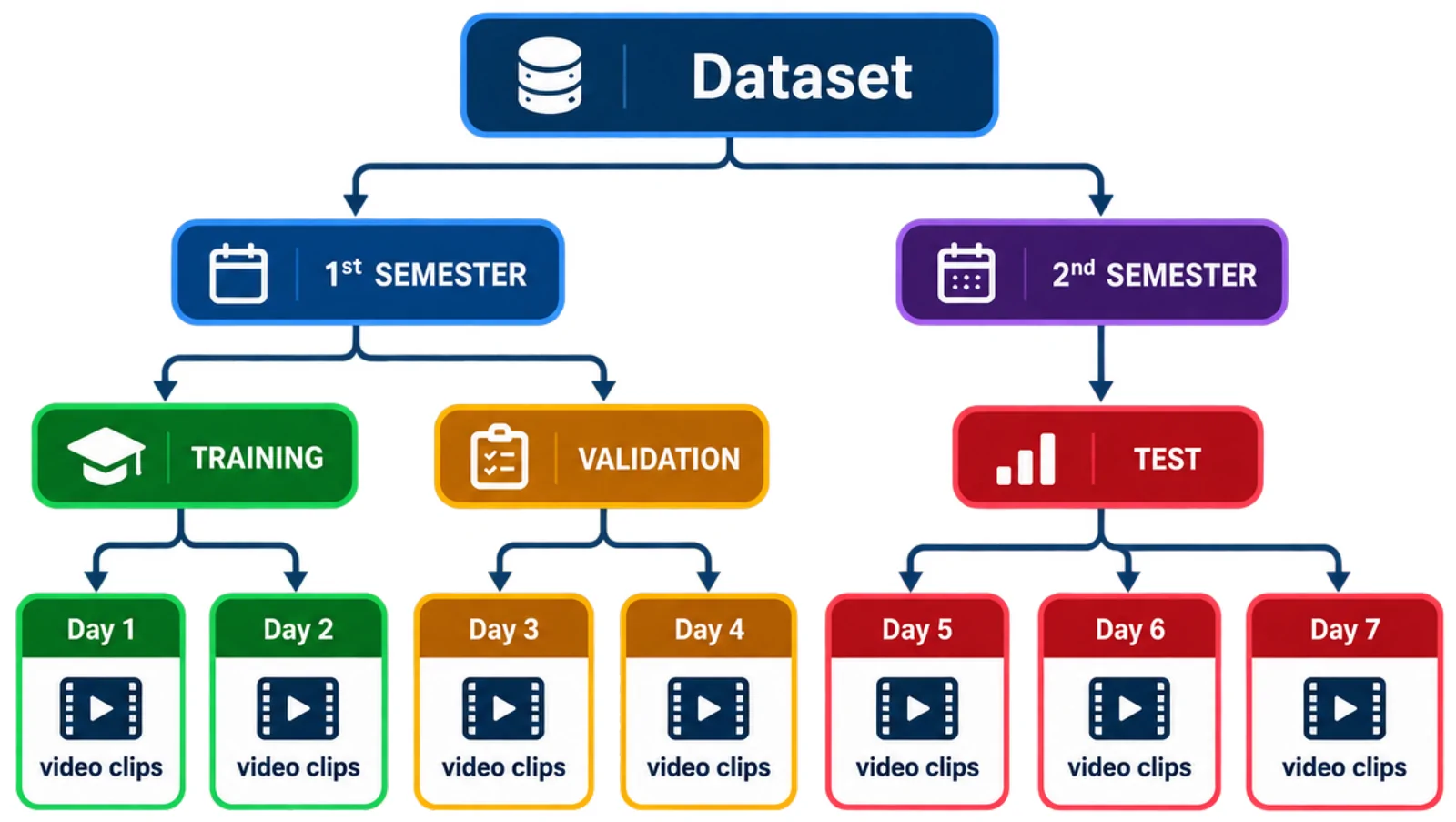
Dataset organization and splitting strategy, illustrating the process of separating video clips by semester and by day.

Since the dataset was constructed from recordings of classes conducted over two semesters, with each recording occurring on a different day, it was decided to divide the data from the first semester into training and validation sets based on the recording days. Before this division, the impact of this choice was evaluated, and it was found that students generally alter their positions and clothing on different class days, which contributes to diversifying the dataset. A total of 80 unique students are represented in the dataset. The recordings were separated according to recording days and semesters to reduce temporal data leakage. However, the dataset is not strictly subject-disjoint. Students included in the training set may also be present in the validation set, and 14 students are shared between the training and test sets. Furthermore, the videos used for training cannot belong to the same days as the validation and test sets, to avoid any form of data leakage. A limitation of this approach is that the remaining video clips from the validation set cannot be used for the training set, to prevent data leakage. As the recordings from the second semester were significantly smaller in quantity than those from the first, it was decided to utilize the entire set of videos from the second semester as the test set; Figure 6 illustrates the data splitting strategy.

Table 2 presents the number of video clips per class for the frontal and lateral cameras, as well as for the entire dataset (i.e., the sum of both). The training dataset comprises six action classes, with between 1578 and 2995 video clips for the lateral view and between 1920 and 2779 video clips for the frontal view. The separation of data by day limited the growth of the training dataset to values above 3000. Excess data from a day assigned to validation and testing could not be used for training, to avoid data leakage. Furthermore, it was decided to train the model using a dataset with a more uniform distribution among the classes; classes such as on computer have a larger dataset, as can be seen in Figure 5, but adding more videos would unbalance the dataset. This can be seen for the reading class in the Lateral set, where the maximum was 1578 videos for the class. The total number of video clips presented in Table 2 is 20,166 for the frontal cameras and 21,870 for the lateral cameras, resulting in a total of 42,036 video clips. These values are lower than the 101,346 video clips reported in Section 3.4 due to the reduction in the number of classes, for which only six classes were selected, as presented in Table 2. In addition, the remaining data, corresponding to the days allocated to the validation and test sets, could not be incorporated into the training set and, consequently, were not used. Video clips in which the student’s actions could not be reliably determined, due to severe occlusion in crowded classroom environments, were also excluded at this stage. However, the number of clips excluded for this reason was not quantified.

**Figure 7:**
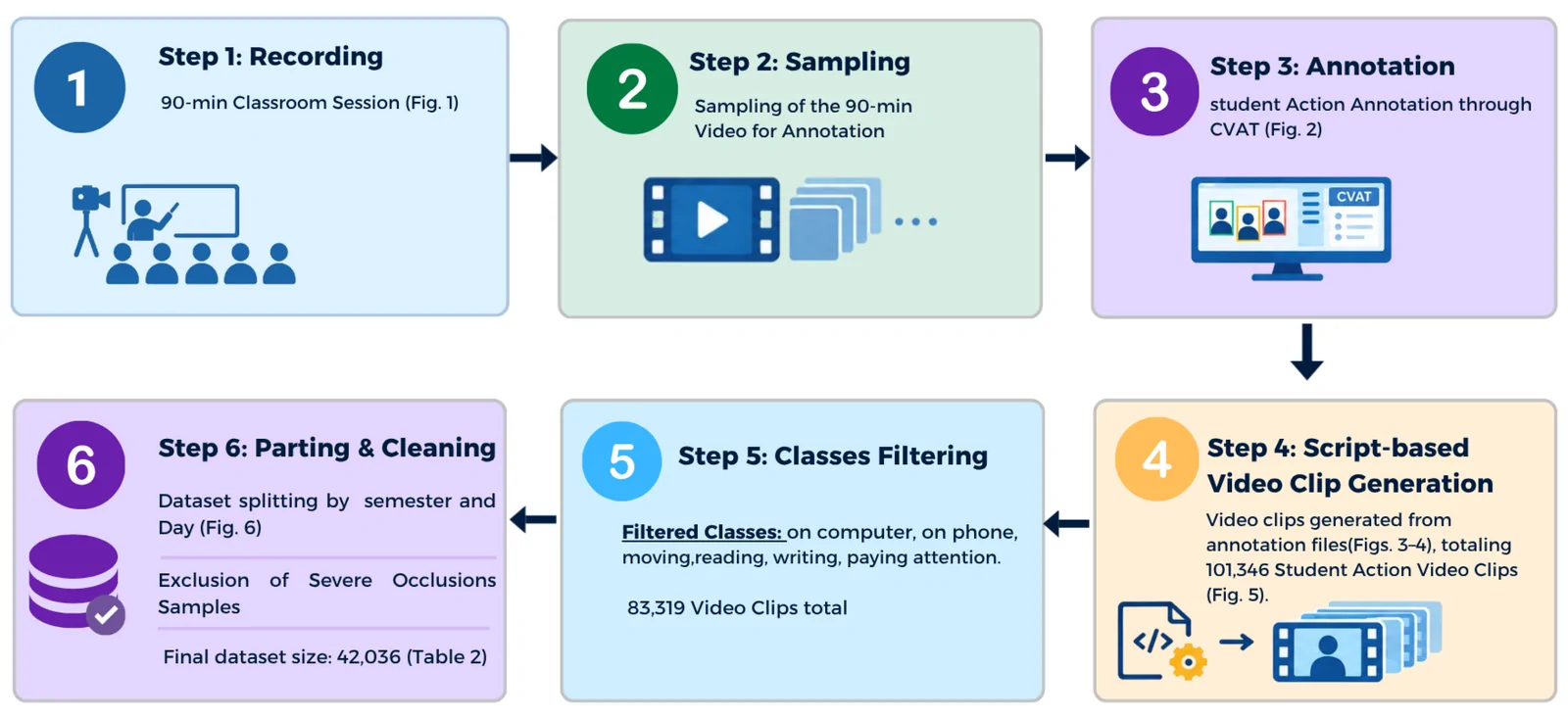
Dataset creation pipeline: from a 90 min classroom recording to the final dataset of 42,036 student action video clips across six action classes.

Figure 7 summarizes the overall dataset construction process, from classroom recording and class annotation to the final dataset. Initially, a 90 min classroom session was recorded (Step 1) and subsequently sampled for annotation (Step 2). Student actions were then annotated using CVAT (Step 3). A custom script was then executed to crop individual student actions, generating an initial set of 101,346 video clips (Step 4). Next, the dataset was filtered to retain only the six target action classes (on computer, on phone, moving, reading, writing, and paying attention), reducing the volume to 83,319 clips (Step 5). Finally, the clips were split into lateral and frontal camera views, partitioned into train, validation, and test sets organized by semester and day, and cleaned by excluding instances with severe occlusion (Step 6). This process yielded a final experimental subset of 42,036 video clips.

### 3.6. Predictive Models and Experimental Configurations

To train the student action classification model, state-of-the-art pre-trained video classification networks were utilized, such as *SlowFast*, *X3D*, *Swin3D* (Swin Transformer for Video), and *MViT*. These four architectures exhibit distinct characteristics that can be leveraged within this dataset. The *SlowFast* network is capable of capturing motion patterns at different temporal rates, making it particularly relevant for distinguishing subtle and rapid behaviors [19]. The *X3D* model offers high computational efficiency, allowing for evaluation of whether competitive accuracy can be achieved using a lightweight architecture [20]. *Swin3D* was selected as a representative 3D Transformer architecture, leveraging hierarchical shifted-window self-attention to model spatial and temporal dependencies [21]. Finally, *MViT* was considered due to its multiscale representation capabilities, which are particularly relevant to classroom behaviors that may occur across different spatial and temporal scales [22]. These models were pre-trained on the Kinetics-400 dataset, which contains 400 human action classes with 400 videos per class [23]. This choice of pre-trained networks facilitates the application of techniques like fine-tuning, which is a beneficial way to obtain good models for custom datasets, both to avoid significant computational complexity.

Table 3 describes the parameters of each model, such as clip size and resolution, in the training step, and the standard clip and resolution of each model. The *Slowfast* models have a different architecture; it utilizes two input streams: a slowpath with 8 frames and a fastpath with 32.

Each video clip in the dataset comprises 64 consecutive frames. However, as shown in Table 3, the architectures under evaluation impose different temporal input requirements: 16 frames for MViT and X3D, 32 frames for Swin3D-S, and 32 and 8 frames for the slow and fast pathways of SlowFast, respectively. To accommodate these differences, temporal sampling of the original 64-frame clips is required, ensuring that the dataset samples conform to the input specifications of each architecture. To this end, the *UniformTemporalSubsample* transformation from the PyTorchVideo library [24] is employed, which uniformly selects the required number of frames from the original clip. This procedure preserves the temporal coverage of the full clip while reducing the number of frames processed by the network. In the case of SlowFast, whose two temporal pathways operate at different sampling rates, the *PackPathway* transformation [25] is applied to distribute the sampled frames between the slow and fast pathways according to the input configuration of the architecture.

In the experiments conducted, pre-trained networks on the Kinetics-400 dataset were utilized, using 20 epochs, and the parameters were tested: learning rate, weight decay, and the alpha of the data augmentation mixup method. The AdamW algorithm was used for optimization. The Cross-Entropy Function with label smoothing with a value of 0.1 was used as the loss function; the Cross-Entropy Function is a function that penalizes incorrect predictions based on confidence and can be used for both binary classifications and multiclass problems, as is the case with the proposed dataset. The data augmentation techniques used were random horizontal flip, MixUp [26], and random erasing. Other data augmentation techniques such as Color Jitter and CutMix were also tested, but did not improve training results. A random seed was also applied, with a value of 42, both for the random of numpy and for cuda through the torch library.

A linear warm-up was applied at the beginning of training, in which the learning rate is gradually increased at each batch until the maximum defined value is reached. This strategy stabilizes the start of training and avoids abrupt updates of the optimizer. After the warm-up period, cosine decay is used to adjust the learning rate over the remaining epochs.

Table 4 shows the tested parameters and networks tested in the exploratory training analysis of the networks for the proposed dataset. In Mixup, the alpha parameter controls the degree of data mixing. Low values (near 0) result in minimal mixing, effectively showing the model’s original images, while high values (near 1) give equal weight to combining two images. To assess this effect, alpha values of 0.4 and 0.8 were tested, both within the standard 0–1 range. An exploratory analysis was also conducted for the warm-up phase. The tested values were 3 and 5. A warm-up value of 3 yields a faster adaptation of the learning rate, while a 5-epoch warm-up provides a more gradual transition. Two initial learning rate values were evaluated in the grid search, $2\times 10^{-5}$ and $5\times 10^{-5}$, together with warm-up periods of 3 and 5 epochs. The learning rate was progressively reduced using a cosine annealing schedule to a minimum value of $1\times 10^{-6}$. Dropout regularization was also evaluated using rates of 0.0, 0.2, and 0.4 (see Table 4), where a rate of 0.0 indicates that dropout was not applied. The *Swin Transformer* was the only model used with a batch size of 32 in the experiments, due to hardware memory limitations that prevented a batch size of 64. To account for the variability associated with the training process, each model was trained and evaluated four times using different random seeds (42, 123, 456, and 789), with the best hyperparameter configuration identified during the grid search.

As illustrated in Figure 1, four different cameras were installed in the classroom, two lateral and two frontal. During the training process, a strategy was applied to train the networks using the data from the frontal and lateral cameras separately in order to evaluate the performance of the training for both the frontal and lateral parts of the classroom. Finally, a training run was performed using the entire dataset based on the best parameters found, as presented in Table 4.

An NVIDIA HGX H200 was used for training and model building in an HPC environment. Pre-trained networks provided by the Pytorch library were utilized; these models are pre-trained using the Kinetics-400 dataset.

### 3.7. Evaluation Metrics

To rigorously assess the performance of the action classification models, we utilize a set of standard metrics derived from the confusion matrix. First, the fundamental variables were defined:

- True Positives (TP): Correctly predicted instances of a specific action class.
- True Negatives (TN): Correctly predicted instances of all other classes.
- False Positives (FP): Instances where the model incorrectly predicted a class.
- False Negatives (FN): Instances where the model missed a true class.
- Total Positives (*P*): The sum of all actual positive instances (TP+FN).
- Total Negatives (*N*): The sum of all actual negative instances (TN+FP).

While accuracy provides a general overview of performance, it can be misleading in imbalanced datasets. Therefore, the F1-score was prioritized, which provides a harmonic mean of precision (the ability to avoid false alarms) and Recall (the ability to detect all relevant instances); as the task involves multiclass classification, the macro F1-score was adopted to give equal importance to the performance of each class, Formula (5). Finally, the Area Under the Curve (AUC) is employed to evaluate the model’s discriminative capability across different thresholds, facilitating a robust comparison between various architectures. In this paper, Formula (6) was used, which calculates an average of single-class measures. The mathematical formulations for these metrics are as follows:

(1) $$Accuracy=\frac{TP+TN}{P+N},$$

(2) $$Precision=\frac{TP}{TP+FP},$$

(3) $$Recall=\frac{TP}{TP+FN},$$

(4) $$F_{1,i}=2\cdot \frac{{\mathrm{Precision}}_{i}\cdot {\mathrm{Recall}}_{i}}{{\mathrm{Precision}}_{i}+{\mathrm{Recall}}_{i}},$$

(5) $$F_{1\text{-}\mathrm{macro}}=\frac{1}{N}\sum_{i=1}^{N}F_{1,i},$$

(6) $$AUC=\frac{\sum_{i=1}^{\left|C\right|}{\mathrm{AUC}}_{i}}{\left|C\right|},$$

where TPR (True Positive Rate) is synonymous with recall, and FPR (False Positive Rate) is defined as $\frac{FP}{FP+TN}$.

The Receiver Operating Characteristic (ROC) curve is a graphical plot that illustrates the diagnostic ability of a binary classifier system as its discrimination threshold is varied. The curve is created by plotting the True Positive Rate (TPR) against the False Positive Rate (FPR) at various threshold settings.

- TPR (Sensitivity): Measures the proportion of actual student actions that were correctly identified.
- FPR (1−Specificity): Measures the proportion of instances from other student action classes that were incorrectly classified as the target action.

The AUC (Area Under the Curve) provides an aggregate measure of performance across all possible classification thresholds. An AUC of 1.0 represents a perfect model, while an AUC of 0.5 indicates a model that performs no better than random guessing. In the context of this study, a high AUC value in the results demonstrates that the model can effectively distinguish between subtle differences among student actions, regardless of the specific probability threshold used.

## 4. Results

This section presents the results of the conducted training. Three different trainings were performed: a first training exploring the front camera dataset, a second exploring the lateral camera dataset, and a third training with all the data from the front and lateral cameras together. For these three trainings performed, the models from Table 3 and all the parameters from Table 4 were tested in order to find the best parameters for each model.

### 4.1. Training for Frontal Camera

In the hyperparameter optimization process, presented in Table 4, the F1 metric was adopted as the main criterion for selecting the best-performing models during training. From these analyses, the performance metrics for each evaluated architecture were obtained. Table 5 shows the best parameters that were obtained on exploratory analysis; only *SlowFast-R101* performed better for a learning rate of $2\times 10^{-5}$, and *MViTv1-b* performed better only for a dropout of 0.4. For Mixup, *Swin3D-S* and *SlowFast-R101* performed better with an α of 0.8.

Table 6 presents the classification performance metrics for the test dataset, accuracy, F1-score macro, and Area Under the Receiver Operating Characteristic Curve (AUC-ROC), achieved by each model following hyperparameter optimization. As previously indicated, the F1 metric was prioritized in the model selection process due to its balanced assessment of precision and recall. The results demonstrate that the *MViTv2-S* architecture showed the highest average performance across all evaluated metrics. The model achieved an accuracy of 74.44% ± 0.45, an F1-score macro of 72.79% ± 0.63, and an AUC-ROC of 84.71% ± 0.26, representing a consistent improvement over all other tested architectures.

The improvements observed with *MViTv2-S* are notable. For instance, the increase in accuracy compared to *MViTv1-b* (71.44 ± 0.93) suggests that the introduction of decomposed relative positional embeddings and residual pooling connections, which significantly enhance spatial–temporal modeling and information flow, contributed to more effective feature extraction and classification. Similarly, the higher macro F1-score demonstrates improved capability in identifying relevant instances while minimizing false positives. The value of the AUC-ROC score (84.71% ± 0.26) underscores the enhanced ability of *MViTv2-S* to discriminate between different video classes.

The *Swin3D-S* model showed competitive performance, achieving an accuracy of 72.86% ± 1.21, although it fell short of *MViTv2-S* across all metrics. The comparatively lower performance of *SlowFast-R101* and *X3D-M* (accuracy in the range of 70–71%) may be attributed to architectures based on 3D convolutions that rely mainly on local spatiotemporal receptive fields, which limit their ability to capture long-range temporal dependencies and global contextual information. It is worth noting that despite a slightly lower accuracy of 70.72% ± 0.60 for X3D-M, this network has a low GFLOP value of 6.7 and 3.9 M parameters (Table 3), showing a good trade-off between accuracy and computational complexity that could support near-real-time performance. These results are consistent with previous findings highlighting the strengths of transformer-based architectures in video understanding, as demonstrated by the high performance of *MViTv2-S* across the evaluated models.

**Figure 8:**
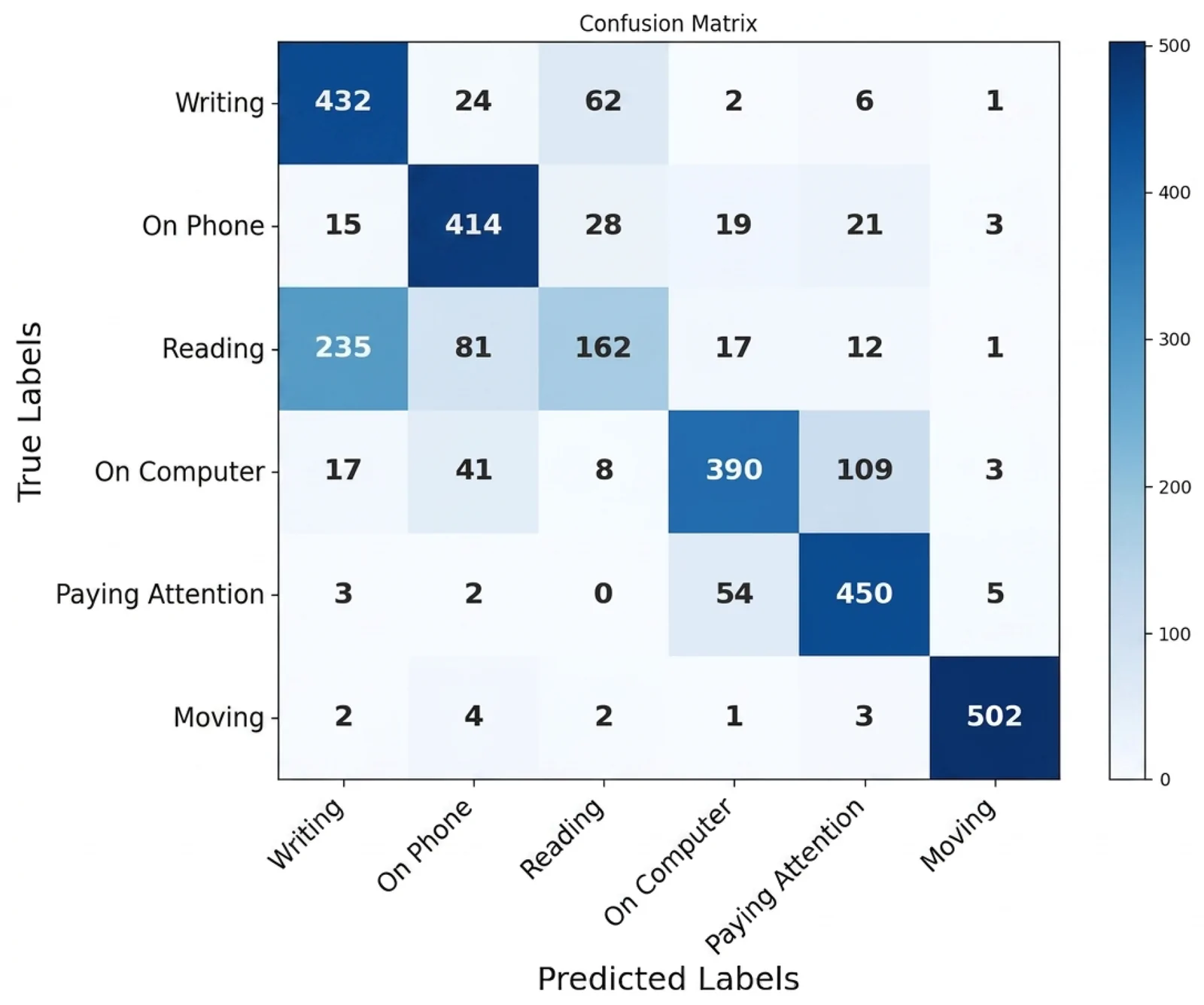
Confusion matrix of the MViTv2-S model (best run) on the frontal-camera test set.

**Figure 9:**
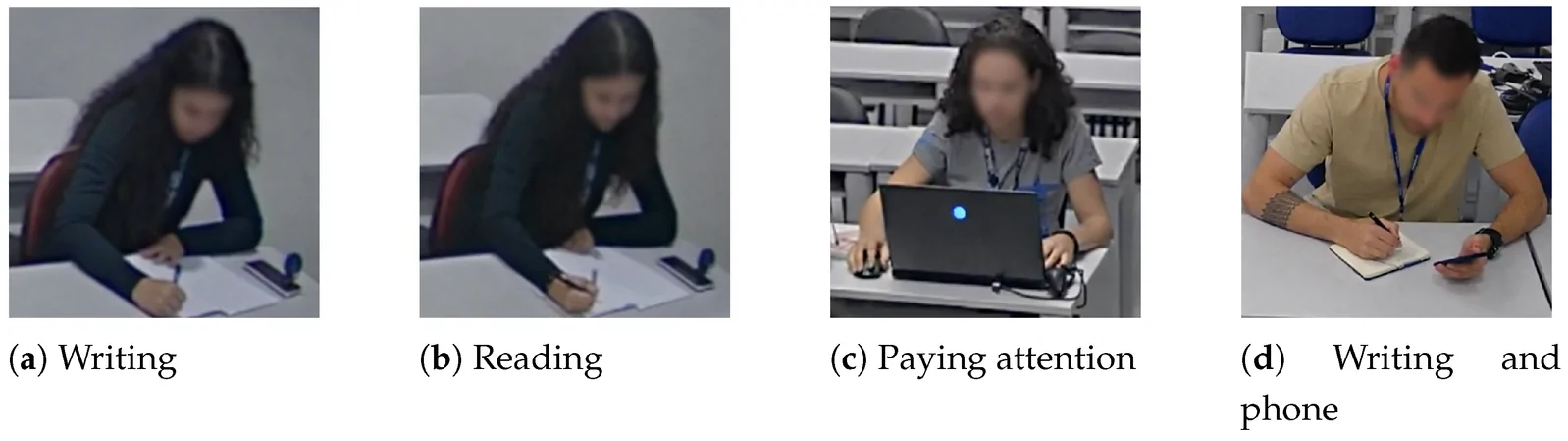
A frame sample of a video clip from some classes that presents ambiguity.

The confusion matrix for the *MViTv2-S* model (Figure 8), derived from the frontal camera view, reveals specific inter-class ambiguities. A consistent pattern of misclassification was observed between the writing and reading categories, as well as between paying attention and on computer. Figure 9 illustrates one frame from each video clip that show the ambiguity case between the classes (a) writing and (b) reading. In the former, the pencil makes contact with the paper, whereas in the latter, it does not. Figure 9c shows a student who is paying attention while still exhibiting characteristics associated with computer use. In the proposed dataset, most students use computers during classes due to their necessity in certain subjects, such as Algorithms. Figure 9d shows another student writing with a phone in his hand, which can cause ambiguity for the model.

The overlap between writing and reading is primarily attributed to the temporal fluidity of these actions; students frequently alternate between these tasks, resulting in highly similar motion signatures. Similarly, the proximity between paying attention and on computer stems from the use of laptops for note-taking during theoretical lectures. In these instances, the sedentary postural patterns are nearly identical, challenging the model’s ability to distinguish between digital and non-digital behaviors.

In contrast, the moving class achieved high precision. The distinct standing postures and high-velocity motion features provided a clear discriminatory signal, mitigating the similarity issues observed among the seated, desk-bound activities.

### 4.2. Training for Lateral Cameras

**Figure 10:**
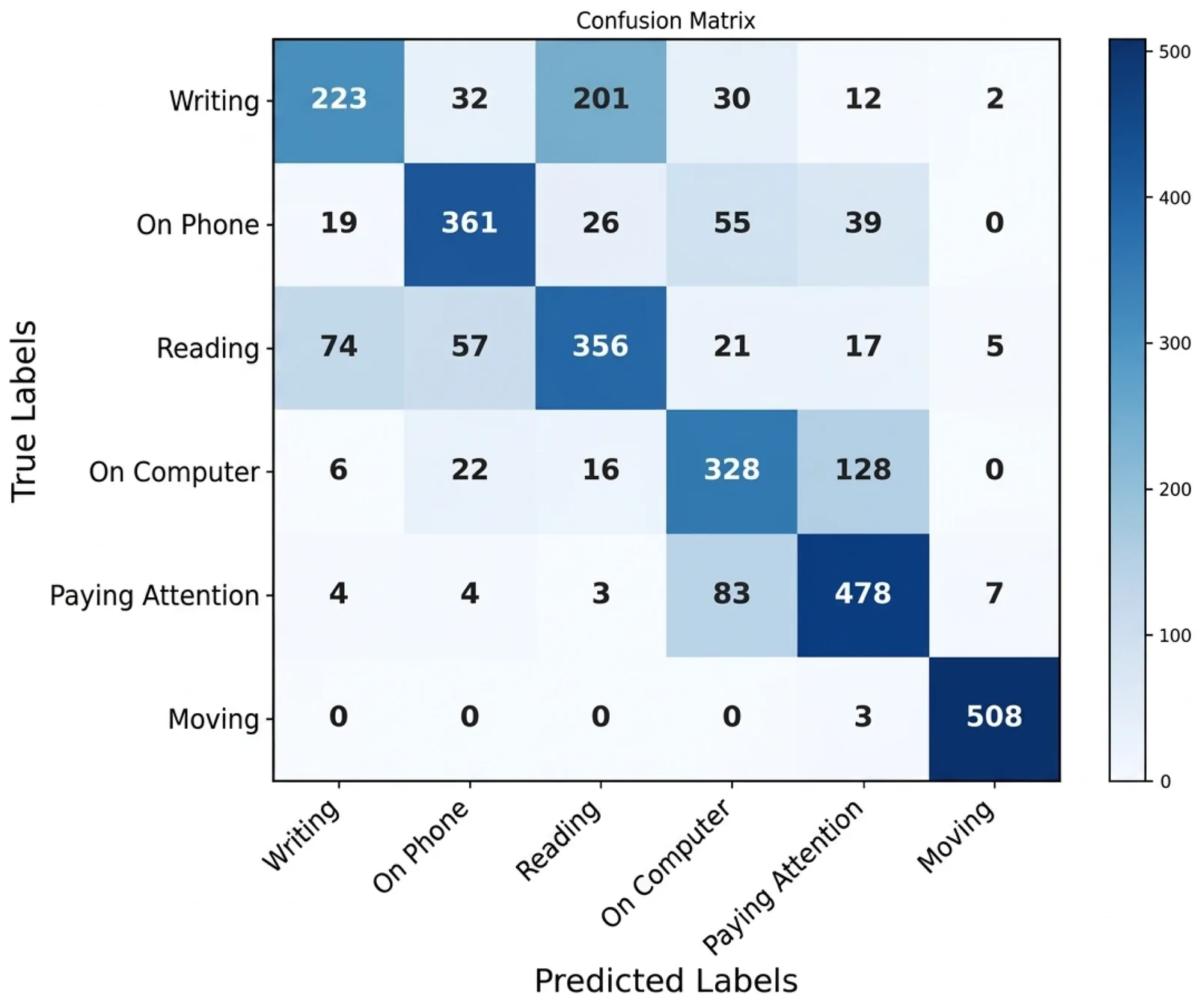
Confusion matrix of the MViT V2 model (best run) on the lateral-camera test set.

The best parameters for the lateral cameras, as presented in Table 7 and Table 8, further reveal the model’s behavior under different perspectives. It is observed that the *MViTv2-S* network also achieved better results for the lateral views for the three metrics analyzed. For the lateral cameras, a decrease in performance was also observed for the *SlowFast-R101* and *X3D-M* networks, with accuracy values of 66.02%±2.54 and 64.01%±1.22, and macro F1-scores of 64.67%±3.18 and 62.26%±1.72, respectively. Analyzing the confusion matrix of the lateral cameras, presented in Figure 10, reveals a distinct behavior in the network’s learning. The confusion between the reading and writing classes persists, with the model exhibiting greater precision in the reading class. The latter class showed a significant increase in recall. On the other hand, the writing class registered a decrease in recall, with most predictions being erroneously directed toward the reading class. Furthermore, the on computer and paying attention classes also maintained patterns of confusion, with 25.6% of the data from the on computer class being incorrectly classified as paying attention.

The observed patterns suggest that the lateral viewpoint, while offering different perspectives, does not fundamentally resolve the challenges of distinguishing between similar actions. The continued confusion between reading and writing, despite the change in viewpoint, reinforces the complexity of these activities and the need for more sophisticated features to differentiate them. The persistence of the on computer and paying attention confusion further supports the idea that postural similarities are a significant factor in model performance.

### 4.3. Training for the Entire Dataset

For training on the complete dataset, which includes both frontal and lateral camera videos clips, the parameters that yielded the best results in the individual training runs (frontal and lateral cameras) were adopted. Table 9 presents the results obtained from training on the complete dataset using data from both cameras. The *MViTv2-S* network maintained its highest average performance compared to the other networks tested. When evaluating the F1 metric, it was observed that the *MViTv2-S* network presented an increase in results when data from both sides of the cameras were considered.

**Figure 11:**
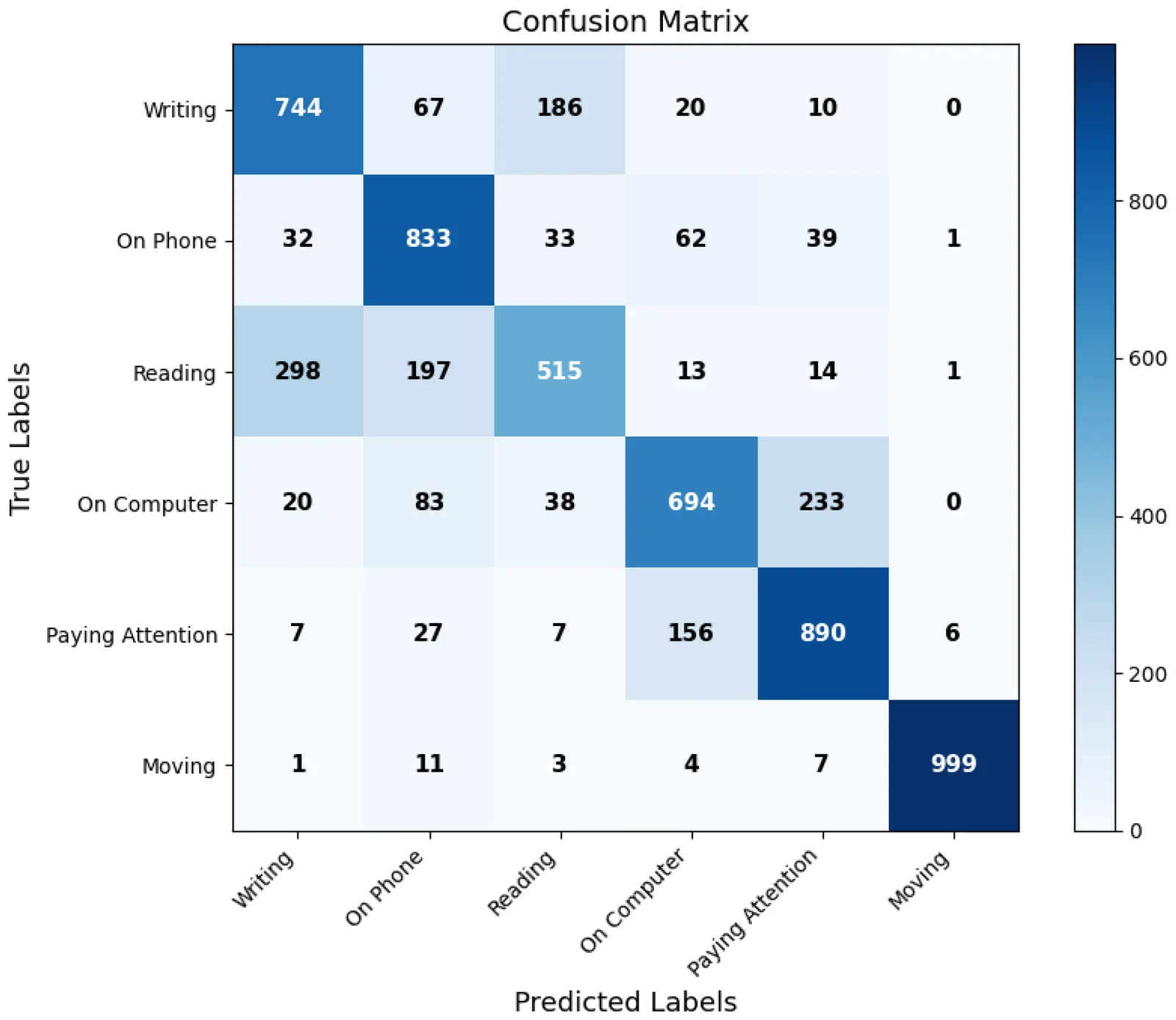
Confusion matrix of the MViT V2 model (best run) on the test set of the combined lateral and frontal cameras.

The analysis of the confusion matrix, presented in Figure 11, referring to the complete dataset training, reveals a similarity with the results obtained previously for the frontal cameras. Consistent patterns remain, such as the confusion between the writing and reading classes, as well as the confusion between the paying attention and on computer classes. The writing class maintained a recall of 72.24%, a reduction compared to the results previously obtained in Figure 8. This decrease can be attributed to the confusion observed in the lateral camera confusion matrix.

This result reinforces the robustness of the *MViTv2-S* model for applications aimed at assessing a student’s action in the classroom, even when considering the integration of data from multiple cameras. The *SlowFast-R101* and *X3D-M* models achieved values below 70% compared to other transformer-based models. Given the cost–benefit ratio, the *X3D-M* model benefits in this comparative analysis compared to *SlowFast-R101* due to the network being lighter, which suggests a lower computational overhead that could facilitate near-real-time performance.

Table 10 numerically shows the results for the above training runs, calculating the recall and precision for each class for the front, lateral, and total cameras, for the best running results. It is interesting to observe that for the on computer class, despite recall being below 70%, precision was 80%, indicating that of the video clips classified as on computer, 80% were actually of that class, unlike paying attention, which had a lower precision, showing that it is receiving a higher number of false positives. The same occurs for the writing class in the front camera. Another interesting point to note is that, when comparing the front and lateral camera configurations, higher recall values are more frequently observed for the front camera. However, this difference should be interpreted with caution, since the front and lateral datasets do not have identical sample sizes or class distributions. Therefore, the observed performance difference cannot be attributed exclusively to the camera viewpoint. Under the conditions of the present dataset and experimental setting, the front camera configuration achieved comparatively higher recall. For the total set, a slight gain in recall for the on phone class is observed, which indicates that the model learned slightly more to classify that class.

Table 11 presents the results of *MViTv2-S* trained on the complete dataset, composed of the frontal and lateral views, and evaluated separately on each test set. For the frontal camera test set, the model trained with the combined data achieved a mean accuracy of 74.13% ± 1.53, a value close to the 74.44% ± 0.45 obtained by the model trained exclusively with frontal camera data. In addition, the macro F1-score showed a small positive variation, going from 72.79% ± 0.63 to 73.16% ± 1.34, as shown in Table 6. These results indicate that the inclusion of lateral camera data did not significantly impair action recognition in the frontal view, preserving the performance obtained with the single-view configuration.

For the lateral camera test set, however, more consistent gains were observed with training using both perspectives. The mean accuracy increased from 70.88% ± 1.04, obtained with the model trained exclusively with lateral data, to 72.92% ± 1.38 when training on the complete dataset. Likewise, the macro F1-score increased from 70.17% ± 1.10 to 72.70% ± 1.40, while the AUC-ROC rose from 82.34% ± 0.64 to 83.75% ± 0.79, as shown in Table 8. The improvement observed in the lateral view suggests that the incorporation of examples from the frontal camera provides complementary visual information that may contribute to learning the features associated with the actions. Overall, these results indicate that multiple perspectives can contribute to action recognition, especially in the lateral view, without significantly impairing the performance in the frontal view.

### 4.4. Engagement Levels

**Figure 12:**
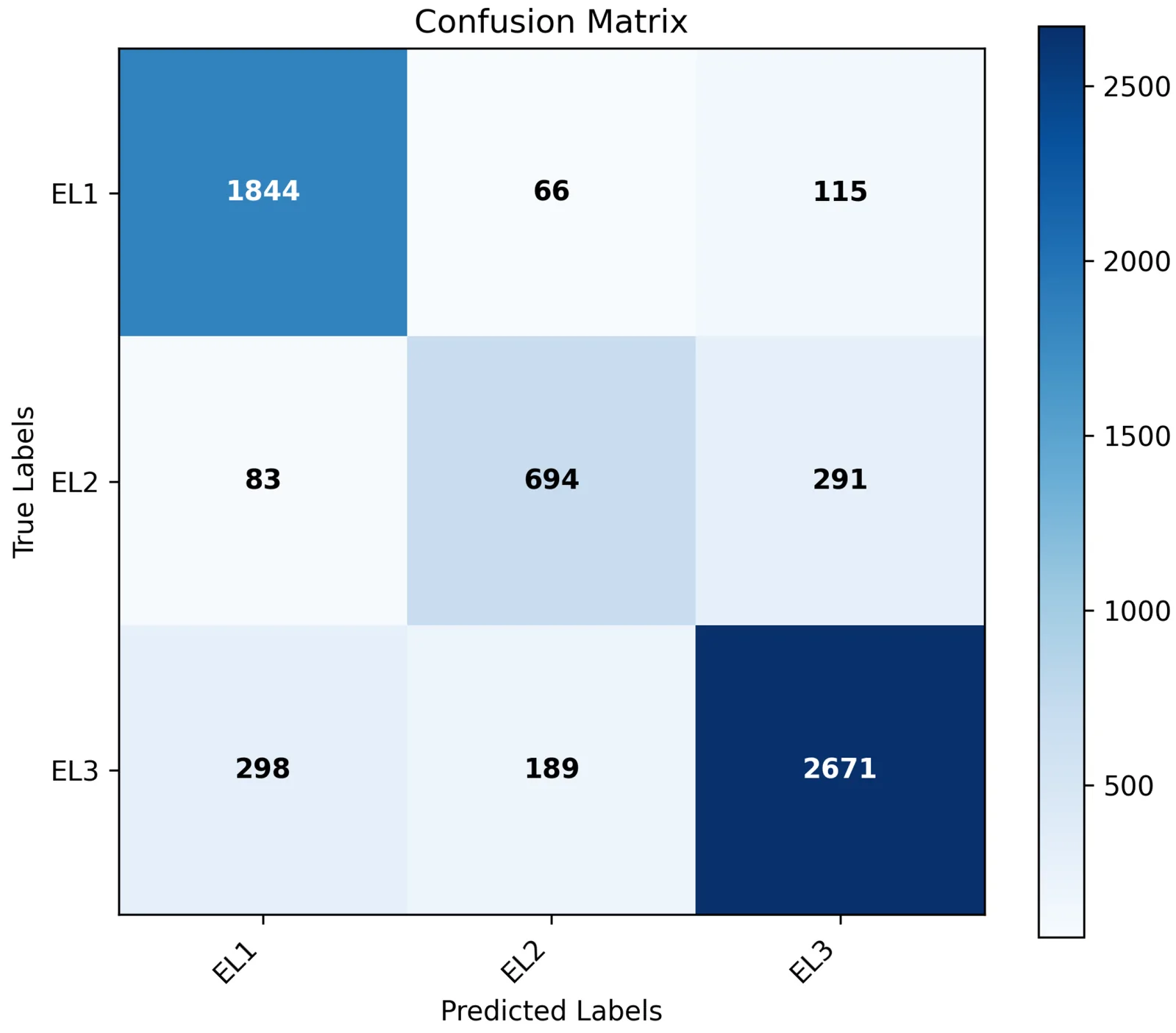
Confusion matrix of the engagement levels (EL1-EL3) for the entire dataset.

It is important to highlight that some real-world applications may be interested only in engagement levels. Therefore, the classes described in this work can be grouped into engagement levels, as described in Section 3.1. Depending on the classroom context and the type of lesson, these classification classes can be regrouped accordingly. Figure 12 shows the resulting confusion matrix for the engagement levels, derived by aggregating the classes of the confusion matrix presented in Figure 11. In addition, the recall, precision, and macro F1-score values for each engagement level are reported in Table 12. Additional experimental results for engagement analysis using frontal and lateral views are provided in the Supplementary Materials.

From the confusion matrix shown in Figure 12, an overall accuracy of 83.33% and a macro F1-score of 80.42% were obtained, indicating good overall classification performance. One of the factors contributing to this accuracy is that the writing and reading classes belong to the same engagement level, EL3. Thus, a student incorrectly classified as reading instead of writing is still assigned to the same engagement level, minimizing the practical impact of this type of confusion. Therefore, confusion between these two actions does not significantly compromise a classroom application aimed at assessing the overall engagement level. EL3 achieved a recall of 84.58% and a precision of 86.81%.

The same does not hold for on computer and paying attention, which belong to different engagement levels. The Medium Engagement (EL2) level, which encompasses the on computer class, achieved a recall of approximately 64.98%. This lower recall is mainly associated with confusion between EL2 and EL3, particularly due to the influence of the paying attention class, with the model presenting a greater difficulty in correctly identifying the Medium Engagement level. In this case, 233 of the 291 samples misclassified as EL3 correspond to paying attention, representing a significant source of confusion between engagement levels. Since EL2 comprises only the on computer class, its recall is effectively identical to the recall of that action class, providing limited additional information beyond the action-level results. Moreover, although this action is mapped to EL2, laptops may also be used for learning-related activities, such as taking notes during lectures. Therefore, the same observable behavior can correspond to different engagement states depending on the classroom context. In the present study, on computer is likely to reflect cognitive effort when students use laptops for note-taking and other class-related activities. However, computer use may also facilitate distraction, particularly when the activity is not directly related to the ongoing lecture. Thus, EL2 engagement estimates should be interpreted in consideration of the instructional context.

The Low Engagement (EL1) class, represented by the moving and on phone classes, achieved a recall of 91.06%, with relatively few misclassifications involving the other engagement levels. This result indicates that the model is highly effective at identifying students exhibiting low engagement behaviors.

### 4.5. Comparison with Related Work

Several studies addressing behavior classification in classrooms employed different methodologies. For example, [10] used the YOLO technique to detect the classes on phone, sleeping, writing, standing, and raising a hand. On the other hand, the work presented by [15] employed the I3D network to classify video clips of actions in classrooms. However, the dataset proposed by this author differs from our proposed dataset because their data covers a broader area of the classroom, including multiple students in a single video clip, while each video clip in our dataset contains only one student. Given these differences, a common point in the results is the recall for moving student classes; [15] reported 89.1% for the *walking in class* class, while our moving class achieved 97% recall.

The work proposed by [14] is very similar to ours regarding the classes; the author’s dataset used the classes listening, talking, reading, writing, playing with mobile phone, and sleeping. For action classification, MSSTGCN was used, as described in Section 2. The accuracy of the model proposed by this author was 87%, whereas the best-performing run of MViTv2-S in this work achieved an accuracy of 75.06% using the frontal camera data. When comparing common classes such as writing and playing with a mobile phone, the recall results for the classes were respectively 90.7% and 92.7% in the mentioned study, and 81.97% and 82.80% in our work, respectively. The listening class proposed by [14], which is similar to our paying attention class, achieved 89.6%, while in our work, paying attention reached 87.55%. On the other hand, the reading class showed a greater discrepancy, with 87.2% recall in the compared study and only 31.89% in our model. The authors of [14] utilized a pose estimation model on their MSSTGCN and suggested that the skeleton information between these two classes exhibited high similarity, making them more likely to be confused. This implies that future works could investigate how pose estimation algorithms can help disambiguate writing and reading classes. Despite the discrepancy in the reading class, other actions such as paying attention, on phone, and writing showed high recall, indicating that they are promising for student behavior analysis. It is important to note that the values compared above are based exclusively on results obtained from the frontal cameras, as the dataset presented by [14] consists solely of images from the front of the classroom.

## 5. Discussion

The creation of a robust dataset for student actions presented a number of significant challenges, impacting both the annotation process and the interpretation of the resulting models’ performance.

While a substantial number of class hours (468 h) were recorded and initially categorized into several student action classes, the complete annotation process proved time-consuming and resource-intensive. A key challenge emerged from the inherent redundancy within the dataset. Due to the structured nature of the academic environment, recurring seating arrangements, invariant visual features, and repetitive postural patterns, considerable overlap was observed across annotated samples. This redundancy, while reflecting the reality of the classroom, complicated the annotation process and raised concerns about potential overfitting.

Beyond the annotation overhead, the physical classroom environment introduced significant technical obstacles. Occlusions caused by students seated in rows frequently hindered clear observation. ’Noise’ from neighboring students, such as overlapping limbs or partial views, complicated the process of capturing the full context of actions. The distance between the students and the camera limits the capture of images with greater detail, making it difficult to identify small objects, such as a pen in a student’s hand. This limitation may negatively impact the discrimination of certain behavioral classes. As a potential improvement, future work may investigate the use of cameras with optical zoom capabilities or higher spatial resolution to capture finer details and potentially enhance the model’s performance. Furthermore, variable distances from the camera, lighting changes, and alterations in student visual appearance contributed to the complexity of the annotation task. The dataset’s scope was further constrained by the fact that it was collected at a single higher education institution in Brazil, limiting the extent to which generalization to other educational environments, particularly those involving younger students and students from different gender and ethnic groups, could impact performance if these variables are relevant to the research question. Notably, certain actions commonly associated with student engagement, such as *raising hand* and sleeping, were observed rarely during class time, rendering them unsuitable for inclusion in the model training process. This scarcity highlights a potential bias in the dataset towards more common behaviors.

In this phase, the model was trained on six prioritized classes that best aligned with the protocol described in Section 3.1. Certain behaviors, such as talking, *raising hands*, and sleeping, were identified and annotated; however, they were excluded from the current training cycle due to an insufficient number of instances. Including these underrepresented classes would have required additional data collection and a larger participant pool to meet performance benchmarks, which fell outside the immediate scope and timeline of this work. Future iterations will focus on expanding these specific datasets to ensure a balanced and robust integration with the existing model.

To mitigate class imbalance and ensure a more balanced representation of the action categories, approximately 2000 video clips were selected for each class from the original 101,346 instances. The resulting per-class counts ranged from 1578 to 2995 (see Table 2). A strict temporal split was implemented for training and validation sets to prevent data leakage and account for daily variations; however, as previously reported, a total of 14 students appear in both the training and test datasets. Although these students are associated with different semesters, the potential influence of this overlap on the results should be considered and further evaluated in future studies. The deliberate inclusion of real-world occlusions and variations was intended to minimize dataset bias and promote broader applicability. However, it is important to acknowledge that recorded classroom hours represent a specific period and may not fully reflect the diversity of behaviors throughout an entire semester or academic year. In addition to these data-related limitations, the annotation protocol also lacks a quantitative measure of inter-annotator agreement, such as Cohen’s k or percentage agreement. Although disagreements were reviewed and resolved by consensus, this prevents a formal assessment of annotation consistency, which should be addressed in future annotation efforts.

Although some datasets are available upon request, direct comparison of results remains limited due to the lack of standardization among the datasets proposed in the literature. Differences in data collection protocols, class definitions, annotation granularity, and capture environments would require scenario-specific adaptations. However, the need to restructure these datasets to fit our problem setting prevented the direct application of our trained model to other datasets, thereby hindering a formal evaluation of the model’s generalization capability.

## 6. Conclusions

This work presented an approach to the construction of a novel dataset and training state-of-the-art computer vision models for video applied in a school environment. The developed dataset distinguishes itself with a real classroom environment featuring undergraduate students at a higher education institution in Brazil. Furthermore, the availability of multiple hours of recorded classes allowed for the expansion of the dataset, capturing different moments from various classes and significantly diversifying the dataset. The utilization of four distinct cameras was fundamental to broaden the angles of capturing student actions, encompassing student interactions from multiple perspectives.

Through the constructed dataset, it was possible to evaluate the performance of five different state-of-the-art neural networks in the task of student behavior recognition. Better performance was observed in networks based on transformers compared to other networks that do not use them, with the *MViTv2-s* network achieving the highest performance in all metrics and in the three proposed scenarios. It was also observed that the camera angle did not affect the performance of some classes, such as paying attention, which maintained a high accuracy rate for both sides of the cameras. However, the lateral cameras for the actions of writing and reading showed a change in confusion, with data from the writing class being more confused with reading compared to the frontal camera. The on computer class had difficulties in achieving higher accuracy due to the similarity with paying attention, as students watched lectures with a notebook on the desk.

Future work will transition this success into a real-time web application, leveraging edge computing and 5G networks to generate actionable insights. Exploring techniques to reduce the inference time of transformer-based models may contribute to faster real-time applications. Additionally, future work will investigate the generalization of these models to diverse environments, such as elementary schools, to further enhance the system’s versatility. Since students often perform overlapping activities, such as writing while using a mobile phone, a multi-label approach will offer an even more precise reflection of complex classroom dynamics. With the advancement of Large Language Models capable of describing situations occurring in videos through text, the application of this type of model can help obtain a more comprehensive description of students’ behavior in the classroom, considering that most existing studies focus on individual student assessment. Future work may further investigate this approach by exploring the potential of these models for broader and more contextualized analyses of the educational environment. Ultimately, this system promises to transform classroom management, fostering more effective and adaptive learning environments.

## Figures and Tables

**Table 1:** Comparative Synthesis of Related Works and the Proposed Study.

Feature	[9]	[10]	[11]	[15]	Our Study
Focus	Eng. Level (Low/Med/High)	Action Detection (HAR)	Teacher/Student Action Recognition	Classroom Behavior (HBR)	Action Recognition and Engagement
Setting/Dataset	CSFED/Public (BAUM-1, etc.)	CStudentAct (Univ. Level)	4500 Videos (8–10 s)	7851 Clips (K-12 level)	42,036 Videos (2.13 s)
Data Capture	Video (Offline/Online)	Pre-recorded videos	Frontal angle recordings	Pre-recorded clips	4-point multicamera
Key Actions	Sleepy, Boredom, Confusion, Focus	Hand, Phone, Sleep, Stand, Write	Board, Hand, Lying, Talking, Tilt	20 Classes (Arguing, etc.)	On Computer, On Phone, Attention, Moving, Writing
Annotation	Facial expressions	Bounding boxes	Temporal frames (1 fps)	Clip-level labels	Continuous spatiotemporal
Architecture	CNN (for FER)	YOLOv5, RT-DETR	SlowFast + MSTA + ETA	I3D-ResNet-50	MViT
Main Contribution	Real-time GEL estimation	Focus on occlusion	Spatial–temporal attention	Large-scale HAR dataset	Multicamera data; Low-intrusive

**Table 2:** Quantities of video clips per class in the two datasets and combined (Entire Dataset).

Class	Frontal Cameras			Lateral Cameras			Entire Dataset		
	Train	Val.	Test	Train	Val.	Test	Train	Val.	Test
On phone	2779	500	500	2802	508	500	5581	1008	1000
Paying Attention	2637	527	514	2995	557	579	5632	1084	1093
Reading	2223	524	508	1578	500	530	3801	1024	1038
Writing	2211	506	527	2692	500	500	4903	1006	1027
Moving	2187	517	514	2597	500	511	4784	1017	1025
On Computer	1920	504	568	2988	533	500	4908	1037	1068
**Total**	**13,957**	**3078**	**3131**	**15,652**	**3098**	**3120**	**29,609**	**6176**	**6251**
**Total video clips: 42,036**									

**Table 3:** Overview of the video recognition architectures evaluated in this work, with their computational cost (GFLOPs), clip length, input resolution, and parameter.

Models	GFLOPs	Clip	Resolution	Params
X3D-M ^1^	∼6.72	16	256×256	3.9 M
MViTv2-S ^2^	∼64.22	16	224×224	34.5 M
MViTv1-b ^3^	∼70.60	16	224×224	36.6 M
SlowFast-R101 ^4^	∼127.20	32 and 8	224×224	62.83 M
Swin3D-S ^5^	∼82.84	32	224×224	49.8 M

^1^ https://pytorch.org/hub/facebookresearch_pytorchvideo_x3d/ (accessed on 27 September 2026). ^2^ https://docs.pytorch.org/vision/main/models/generated/torchvision.models.video.mvit_v2_s.html (accessed on 27 September 2026). ^3^ https://docs.pytorch.org/vision/main/models/generated/torchvision.models.video.mvit_v1_b.html (accessed on 27 September 2026). ^4^ https://pytorch.org/hub/facebookresearch_pytorchvideo_slowfast/ (accessed on 27 September 2026). ^5^ https://docs.pytorch.org/vision/main/models/generated/torchvision.models.video.swin3d_s.html (accessed on 27 September 2026).

**Table 4:** Training hyperparameters used in the activity recognition experiments. When multiple values are listed, all corresponding configurations were explored, and the best-performing combination was selected for the final models.

Parameter	Value
Model	MViTv2-S, MViTv1-B, SlowFast-R101, X3D-M, Swin3D-S
Number of epochs	20
Batch size	32, 64
Learning rate	$2\times 10^{-5}$, $5\times 10^{-5}$
Dropout	0.0, 0.2, 0.4
Optimizer	AdamW
Weight decay	0.05
Scheduler	Cosine Annealing with Warm-up
Warm-up	3, 5 epochs
Loss function	Cross-Entropy
Number of classes	6
Data augmentation	Random Erasing, Horizontal Flip
Mixup (α)	0.4, 0.8
Seed	42, 123, 456, 789

**Table 5:** Best hyperparameter configuration per model in the frontal camera.

Model	Batch	LR	W. Decay	Drop.	Warm.	Mixup
MViTv1-b	64	$5\times 10^{-5}$	0.05	0.4	3	0.4
MViTv2-S	64	$5\times 10^{-5}$	0.05	0.0	5	0.4
Swin3D-S	32	$5\times 10^{-5}$	0.05	0.0	3	0.8
SlowFast-R101	64	$2\times 10^{-5}$	0.05	0.0	5	0.8
X3D-M	64	$5\times 10^{-5}$	0.05	0.0	5	0.4

**Table 6:** Classification algorithm results with the test dataset and use of the best parameters found for the frontal database, with bold values indicating the best results.

Algorithm	Accuracy (%)	Macro F1-Score (%)	AUC-ROC (%)
*MViTv1-b*	71.44 ± 0.93	70.23 ± 2.34	83.01 ± 0.53
*MViTv2-S*	**74.44 ± 0.45**	**72.79 ± 0.63**	**84.71 ± 0.26**
*Swin3D-S*	72.86 ± 1.21	71.00 ± 1.14	83.75 ± 0.71
*SlowFast-R101*	71.59 ± 1.44	69.73 ± 1.74	83.07 ± 0.85
*X3D-M*	70.72 ± 0.60	69.31 ± 0.72	82.41 ± 0.34

**Table 7:** Best hyperparameter configuration per model in the lateral camera.

Model	Batch	LR	W. Decay	Drop.	Warm	Mixup
MViTv1-b	64	$5\times 10^{-5}$	0.05	0.4	5	0.8
MViTv2-S	64	$5\times 10^{-5}$	0.05	0.0	5	0.4
Swin3D-S	32	$2\times 10^{-5}$	0.05	0.0	5	0.8
SlowFast-R101	64	$5\times 10^{-5}$	0.05	0.0	5	0.4
X3D-M	64	$2\times 10^{-5}$	0.05	0.0	3	0.8

**Table 8:** Classification algorithm results with the test dataset and use of the best parameters found for the lateral database, with bold values indicating the best results.

Algorithm	Acc. (%)	Macro F1-Score (%)	AUC-ROC (%)
*MViTv1-b*	68.55 ± 1.89	67.88 ± 2.01	80.94 ± 1.10
*MViTv2-S*	**70.88 ± 1.04**	**70.17 ± 1.10**	**82.34 ± 0.64**
*Swin3D-S*	68.76 ± 1.36	68.17 ± 1.40	81.16 ± 0.78
*SlowFast-R101*	66.02 ± 2.54	64.67 ± 3.18	79.42 ± 1.60
*X3D-M*	64.01 ± 1.22	62.26 ± 1.72	78.19 ± 0.79

**Table 9:** Classification algorithm results for the entire dataset training, with bold values indicating the best results.

Algorithm	Acc. (%)	Macro F1-Score (%)	AUC-ROC (%)
MViTv1-b	73.30 ± 1.01	72.40 ± 0.34	83.66 ± 0.18
*MViTv2-S*	**74.63 ± 0.79**	**74.42 ± 0.89**	**84.82 ± 0.35**
*Swin3D-S*	72.45 ± 1.00	72.20 ± 1.20	83.48 ± 0.59
*SlowFast-R101*	69.14 ± 1.07	68.66 ± 0.121	81.55 ± 0.64
*X3D-M*	67.08 ± 0.45	67.03 ± 0.48	80.30 ± 0.26

**Table 10:** Per-activity recall and precision of the best-performing MViTv2-S model on the entire test set.

Activity	Front (%)		Lateral (%)		Total (%)	
	Recall	Prec.	Recall	Prec.	Recall	Prec.
Writing	81.97	61.36	44.60	68.40	72.44	67.51
On Phone	82.80	73.14	72.20	75.84	83.30	68.39
Reading	31.89	61.83	67.17	59.14	49.61	65.86
On Computer	68.66	80.75	65.60	63.44	64.98	73.13
Paying Attention	87.55	74.88	82.56	70.61	81.43	74.60
Moving	97.67	97.48	99.41	97.32	97.46	99.21

**Table 11:** Leave-one-view-out evaluation of the MViTv2-s model trained on the combined dataset (frontal + lateral) and evaluated separately on each single-view test set.

Test Set	Accuracy (%)	Macro F1-Score (%)	AUC-ROC (%)
Frontal camera	74.13 ± 1.53	73.16 ± 1.34	84.55 ± 0.92
Lateral camera	72.92 ± 1.38	72.70 ± 1.40	83.75 ± 0.79

**Table 12:** Performance metrics for each engagement class, with the overall accuracy for the entire dataset.

Class	Precision (%)	Recall (%)	F1-Score (%)
EL3	86.81	84.58	85.68
EL2	73.13	64.98	68.82
EL1	82.88	91.06	86.78
Accuracy	83.33		
macro F1-score	80.42		

## Data Availability

The dataset that supports the findings of this study is not publicly available, due to student privacy concerns. A new version of the dataset suitable for public release is currently being developed with anonymized faces and will be shared with the community in the future.

## Supplementary Materials

The following supporting information can be downloaded at https://www.mdpi.com/article/10.3390/s26206608/s1, Figure S1: Confusion Matrix of the engagement levels (EL1–EL3) for the lateral-view; Figure S2: Confusion Matrix of the engajament levels (EL1–EL3) for the frontal view; Table S1: Performance metrics for engagement levels (EL1–EL3), with the overall accuracy for the lateral-view; Table S2: Performance metrics for engagement levels (EL1–EL3), with the overall accuracy for the frontal-view.
