# Supplementary material for: A Multicamera Classroom Dataset for Deep Learning-Based Student Action Recognition and Engagement Analysis
Source: Sensors (Basel). 2026 Oct 19;26(20):6608. doi: 10.3390/s26206608 (PMC13709081; doi:10.3390/s26206608)
Supplement: Supplementary file 1 [file sensors-26-06608-s001.zip › sensors-4541462-supplementary.pdf]

Supplementary Materials

Table S1: Performance metrics for engagement levels (EL1-EL3), with the overall accuracy for the lateral-view.

**Class**	**Precision (%)**	**Recall (%)**	**F1-score(%)**
EL3	85.23	85.02	85.13
EL2	63.44	65.60	64.50
EL1	87.07	85.95	86.51
**Accuracy**	82.21		
**F1-score macro**	78.71		

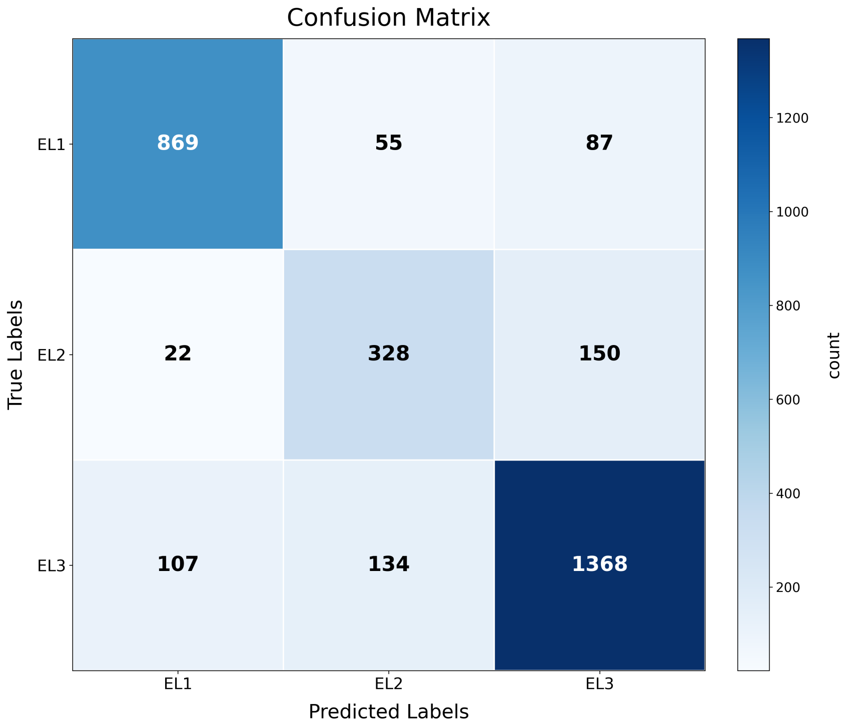

Figure S1: Confusion Matrix of the engagement levels(EL1-EL3) for the lateral-view.

Table S2: Performance metrics for engagement levels (EL1-EL3), with the overall accuracy for the frontal-view

**Class**	**Precision (%)**	**Recall (%)**	**F1-score(%)**
EL3	86.92	87.93	87.42
EL2	80.75	68.66	74.22
EL1	85.38	91.03	88.11
**Accuracy**	85.44		
**F1-score macro**	83.25		

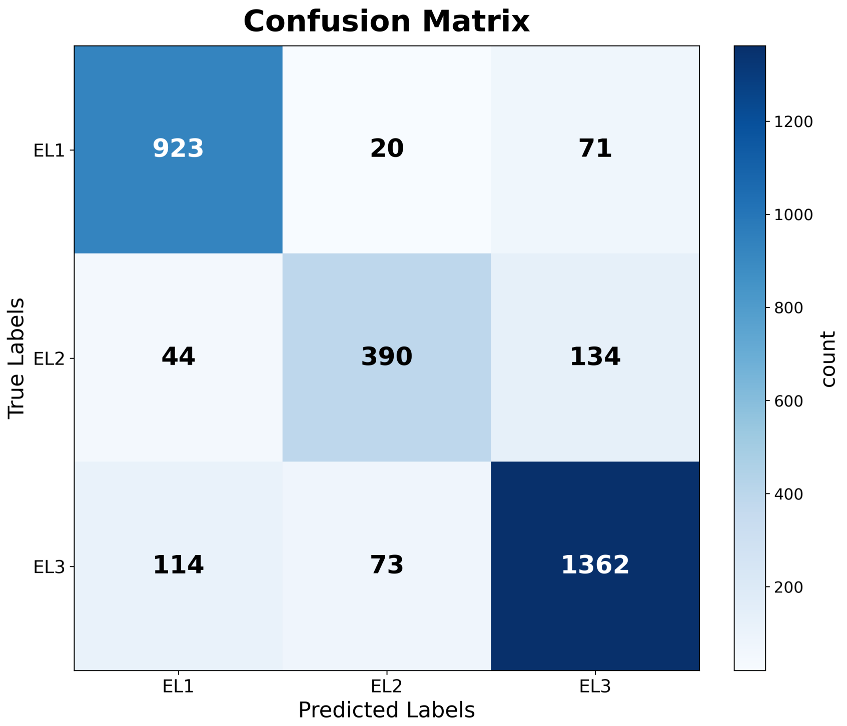

Figure S2: Confusion Matrix of the engajament levels(EL1-EL3) for the frontal view.
